# Noncoding RNA *Terc-53* and hyaluronan receptor Hmmr regulate aging in mice

**DOI:** 10.1093/procel/pwae023

**Published:** 2024-05-09

**Authors:** Sipeng Wu, Yiqi Cai, Lixiao Zhang, Xiang Li, Xu Liu, Guangkeng Zhou, Hongdi Luo, Renjian Li, Yujia Huo, Zhirong Zhang, Siyi Chen, Jinliang Huang, Jiahao Shi, Shanwei Ding, Zhe Sun, Zizhuo Zhou, Pengcheng Wang, Geng Wang

**Affiliations:** State Key Laboratory for Cellular Stress Biology, Innovation Center for Cell Signaling Network, School of Life Sciences, Xiamen University, Xiamen 361102, China; State Key Laboratory for Cellular Stress Biology, Innovation Center for Cell Signaling Network, School of Life Sciences, Xiamen University, Xiamen 361102, China; State Key Laboratory for Cellular Stress Biology, Innovation Center for Cell Signaling Network, School of Life Sciences, Xiamen University, Xiamen 361102, China; State Key Laboratory for Cellular Stress Biology, Innovation Center for Cell Signaling Network, School of Life Sciences, Xiamen University, Xiamen 361102, China; State Key Laboratory for Cellular Stress Biology, Innovation Center for Cell Signaling Network, School of Life Sciences, Xiamen University, Xiamen 361102, China; State Key Laboratory for Cellular Stress Biology, Innovation Center for Cell Signaling Network, School of Life Sciences, Xiamen University, Xiamen 361102, China; State Key Laboratory for Cellular Stress Biology, Innovation Center for Cell Signaling Network, School of Life Sciences, Xiamen University, Xiamen 361102, China; State Key Laboratory for Cellular Stress Biology, Innovation Center for Cell Signaling Network, School of Life Sciences, Xiamen University, Xiamen 361102, China; State Key Laboratory for Cellular Stress Biology, Innovation Center for Cell Signaling Network, School of Life Sciences, Xiamen University, Xiamen 361102, China; State Key Laboratory for Cellular Stress Biology, Innovation Center for Cell Signaling Network, School of Life Sciences, Xiamen University, Xiamen 361102, China; State Key Laboratory for Cellular Stress Biology, Innovation Center for Cell Signaling Network, School of Life Sciences, Xiamen University, Xiamen 361102, China; School of Life Sciences, Tsinghua University, Beijing 100084, China; State Key Laboratory for Cellular Stress Biology, Innovation Center for Cell Signaling Network, School of Life Sciences, Xiamen University, Xiamen 361102, China; State Key Laboratory for Cellular Stress Biology, Innovation Center for Cell Signaling Network, School of Life Sciences, Xiamen University, Xiamen 361102, China; State Key Laboratory for Cellular Stress Biology, Innovation Center for Cell Signaling Network, School of Life Sciences, Xiamen University, Xiamen 361102, China; State Key Laboratory for Cellular Stress Biology, Innovation Center for Cell Signaling Network, School of Life Sciences, Xiamen University, Xiamen 361102, China; State Key Laboratory for Cellular Stress Biology, Innovation Center for Cell Signaling Network, School of Life Sciences, Xiamen University, Xiamen 361102, China; State Key Laboratory for Cellular Stress Biology, Innovation Center for Cell Signaling Network, School of Life Sciences, Xiamen University, Xiamen 361102, China

**Keywords:** *TERC-53*, mitochondrial noncoding RNAs, brain aging, neuroinflammation, ubiquitination

## Abstract

One of the basic questions in the aging field is whether there is a fundamental difference between the aging of lower invertebrates and mammals. A major difference between the lower invertebrates and mammals is the abundancy of noncoding RNAs, most of which are not conserved. We have previously identified a noncoding RNA *Terc-53* that is derived from the RNA component of telomerase *Terc*. To study its physiological functions, we generated two transgenic mouse models overexpressing the RNA in wild-type and early-aging *Terc*^−/−^ backgrounds. *Terc-53* mice showed age-related cognition decline and shortened life span, even though no developmental defects or physiological abnormality at an early age was observed, indicating its involvement in normal aging of mammals. Subsequent mechanistic study identified hyaluronan-mediated motility receptor (Hmmr) as the main effector of *Terc-53*. *Terc-53* mediates the degradation of Hmmr, leading to an increase of inflammation in the affected tissues, accelerating organismal aging. adeno-associated virus delivered supplementation of Hmmr in the hippocampus reversed the cognition decline in *Terc-53* transgenic mice. Neither *Terc-53* nor Hmmr has homologs in *C*. *elegans*. Neither do arthropods express hyaluronan. These findings demonstrate the complexity of aging in mammals and open new paths for exploring noncoding RNA and Hmmr as means of treating age-related physical debilities and improving healthspan.

## Introduction

Noncoding RNA-derived RNAs are a group of RNAs that are the processed products of the existing noncoding RNAs. For example, tRNA-derived small RNAs (tsRNAs) can be categorized into at least six types based on the sites of the cleavage on the mature or precursor tRNA transcripts (Gebetsberger and Polacek, 2013). Even though most tsRNAs do not have well-defined functions, some have been shown to play important biological roles in ribosome biogenesis and mitochondrial translation (Kim et al., 2017; Li et al., 2024). Another example of noncoding RNA-derived RNA is mascRNA that is a small tRNA-like RNA derived from a long noncoding RNA *MALAT1* (metastasis-associated lung adenocarcinoma transcript 1) (Wilusz et al., 2008). mascRNA is a cytosolic RNA even though *MALAT1* localizes predominantly in nuclear speckles, and has been shown to promote cytosolic translation by stabilizing QARS (glutaminyl-tRNA synthetase) (Lu et al., 2020; Wilusz et al., 2008). The progress on the functional study of these noncoding RNA-derived RNAs, however, lags behind the discovery of these new RNAs, as it is difficult to exclude the effects of the RNAs from which these smaller RNAs are derived.

We have previously identified a noncoding RNA derived from the RNA component of telomerase *TERC* (Cheng et al., 2018; Greider and Blackburn, 1989; Morin, 1989; Nguyen et al., 2018). We named it *TERC-53* based on the site of cleavage in human (Cheng et al., 2018). The processing happens in mitochondria, but *TERC-53* is exported and resides mostly in the cytosol (Cheng et al., 2018). Its levels in the brain and the liver of mice have been shown to increase as the mice age, peaking at the age of 10 months, and then decrease as they grow older, even though the levels of full-length *Terc* remain the same (Zheng et al., 2019). There has been some evidence suggesting *Terc-53*’s involvement in cognition decline during aging. The evidence, however, is preliminary, and lacks mechanistic explanation (Zheng et al., 2019). Whether the RNA is expressed differentially in different brain regions, or whether there is cell-type specificity functionally is not clear.

There have been reports that both *TERC* and the protein component of telomerase TERT possess some telomerase-independent functions (Cayuela et al., 2005; Lipskaia et al., 2024; Liu et al., 2019). For example, Expression of the catalytically inactive form of Tert or the wild-type protein has been shown to reduce p21 levels in the lungs of the aged mice (Lipskaia et al., 2024). *TERC* is ubiquitously expressed in most terminally differentiated human somatic cells that lack telomerase activity (Yi et al., 1999). In addition, the level of *TERC* is often higher than what is required to form telomerase with TERT in telomerase-positive cells (Blasco et al., 1996; Xi and Cech, 2014). Expression of *TERC* in telomerase-negative cells has been shown to alter the expression of many genes involved in immunity (Liu et al., 2019). There is also evidence supporting telomerase-independent functions of *TERC* in apoptosis, and DNA damage responses (Gazzaniga and Blackburn, 2014; Ting et al., 2009). Despite these findings, the molecular mechanisms of these functions are unclear. *TERC-53*’s involvement has not been considered in most of these studies.

To gain a better understanding of *Terc-53*’s physiological functions and the molecular mechanism, we have established a series of mouse models, and cellular and biochemical systems. Studies on these models and systems have shown that *Terc-53* functions as a molecular scaffold bringing together Hmmr and Trim25, accelerating Hmmr’s ubiquitination-mediated degradation, which consequently leads to neuroinflammation and cognition decline.

## Results

### *Terc-53* regulates organismal aging, independent of *Terc* or telomerase

**Figure 1.**
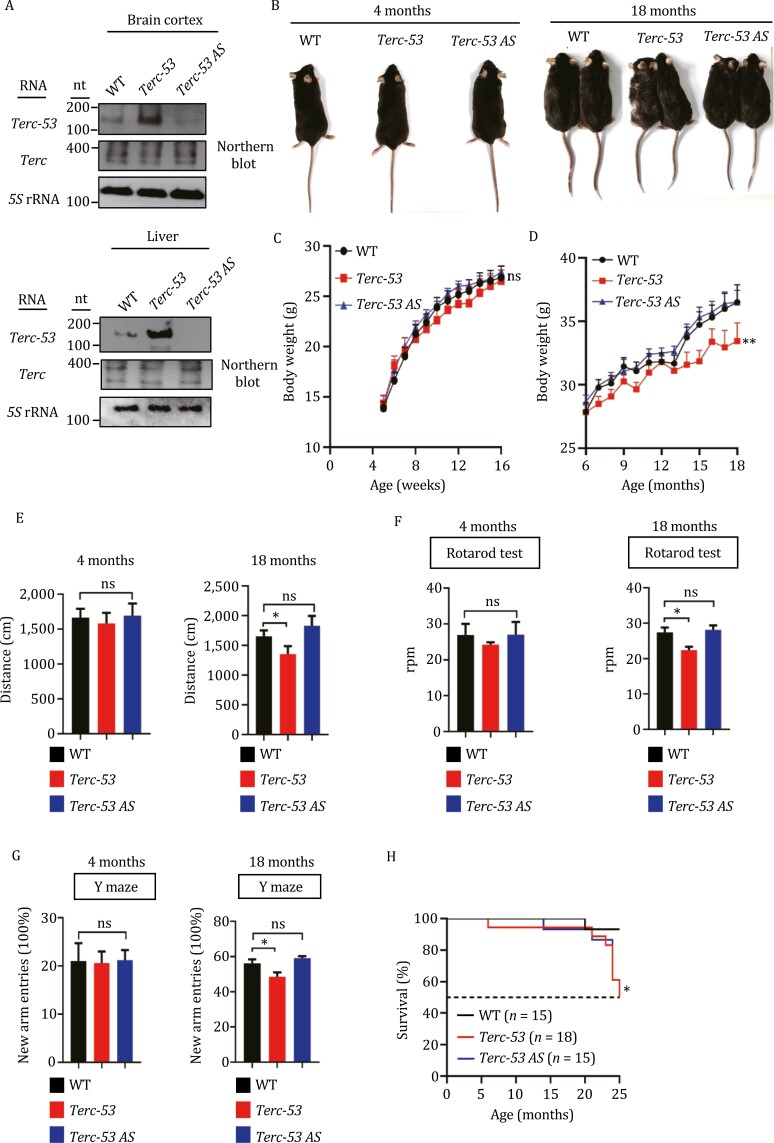
***Terc-53* regulates organismal ageing.** (A) *Terc-53* and *Terc* expression levels in the brain cortex and liver of 3-month-old wild type mice (WT), transgenic *Terc-53-*overexpressing mice (*Terc-53*) and transgenic *Terc-53* antisense-expressing mice (*Terc-53 AS*) were examined by Northern blot. *5S* rRNA was used as a loading control. (B) Representative images of WT, *Terc-53* or *Terc-53 AS* mice at 4 months and 18 months of age. (C) The body weight of WT, *Terc-53* and *Terc-53 AS* from week 5–16. *n* = 8 male mice per group. (D) The body weight of WT, *Terc-53* and *Terc-53 AS* from month 6–18, *n* = 12 (WT male), *n* = 10 (*Terc-53* male) and *n* = 13 (*Terc-53 AS* male). (E) The movement distance of WT, *Terc-53* and *Terc-53 AS* mice in open field test at 4 months and 18 months of age. *n* = 10 male mice per group. (F) The rotarod performance of WT, *Terc-53* and *Terc-53 AS* mice at 4 months and 18 months of age. *n* = 8 male mice per group. (G) Y maze test of WT, *Terc-53* and *Terc-53 AS* mice at 4 months and 18 months of age, *n* = 11 (WT male), *n* = 9 (*Terc-53* male) and *n* = 11 (*Terc-53 AS* male). (H) Cumulative survival curves of WT, *Terc-53* and *Terc-53 AS* mice, *n* = 15 (WT male), *n* = 18 (*Terc-53* male) and *n* = 15 (*Terc-53 AS* male). The *P* value was calculated using a two-tailed log rank test. Data are presented as mean ± SEM. ns, no significant difference, *P < 0.05, **P < 0.01.

To examine whether *Terc-53* is involved in aging, we first examined *Terc-53* levels in existing Alzheimer’s disease models. In a mouse N2a (a mouse neural crest-derived cell line) cell model that stably expresses human APP gene for Aβ production (N2a-APP695), *Terc-53* levels were significantly upregulated, while full-length *Terc* levels remained the same (Fig. S1A and S1B). Similar changes were also observed in the hippocampus of 5× FAD mice at 6 months of age. These results suggest that dysregulation of *Terc-53* levels may also play a role in development of aging-related neurodegenerative diseases (Fig. S1C–E). To examine the potential role of *Terc-53* in organismal aging, two lines of transgenic mice were generated, one overexpressing *Terc-53* and the other expressing *Terc-53* antisense RNA (*Terc-53 AS*), both under the control of U6 promoter (Fig. S2A and S2B). Overexpression of *Terc-53* was detected in the brain cortex and the liver of *Terc-53* mice, while knockdown was achieved in both tissues of *Terc-53 AS* mice (Fig. 1A). The levels of the full-length *Terc* were unchanged in both mice compared to the wild-type control (Fig. 1A). Up until to four months of age, no significant changes of body weight, movement, motor skill (based on rotarod tests), or cognition (based on Y maze test) were observed between *Terc53*, *Terc-53 AS* and wild-type control mice (Fig. 1B–G). Starting from the sixth month, *Terc-53* mice started to show a lagging of growth in body weight compared to *Terc-53 AS* and the wild-type control (Fig. 1B). At 18 months of age, *Terc-53* mice exhibited more severe aging phenotypes in all the tests. The mice were significantly smaller, had more gray hair, moved slower, showed less motor coordination, and more cognition decline (Fig. 1B–G). Unlike most fast-aging mouse models that show developmental problems, mental retardation or increase in the incidence of tumors (Barlow et al., 1996; Chang et al., 2004; Fatkin et al., 1999; Varela et al., 2005), *Terc-53* mice only showed a faster decline of health in general compared to the control mice, a reflection of normal aging. A shorter lifespan was also observed with *Terc-53* mice (Fig. 1H). No significant difference of the aging indexes was observed between *Terc-53 AS* mice and the wild-type control even though *Terc-53* levels were decreased by the antisense RNA (Fig. 1B–H). We will come back to this discrepancy with an explanation of molecular mechanism later in the text.

**Figure 2.**
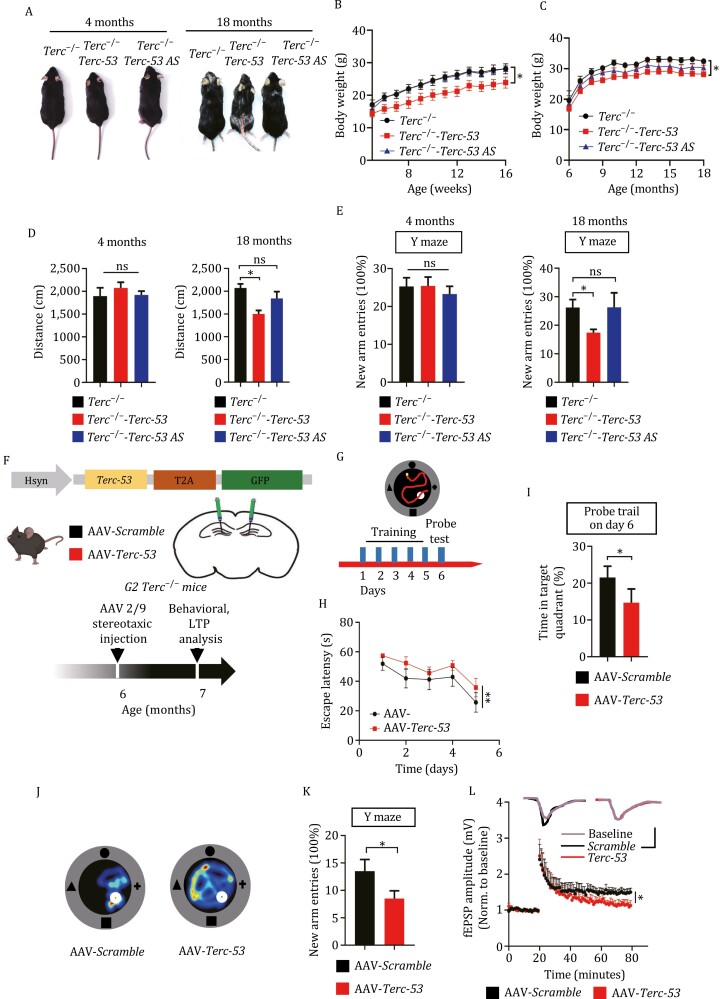
***Terc-53* regulates organismal aging, independent of *Terc*.** (A) Representative images of *Terc* knockout (*Terc*^−/−^), *Terc* knockout, transgenic *Terc-53-* overexpressing mice (*Terc*^−/−^-*Terc-53*) and *Terc* knockout, transgenic *Terc-53* antisense-expressing mice (*Terc*^−/*−*^-*Terc-53 AS*) at 4 months and 18 months of age. (B) The body weight of *Terc*^−/−^ and *Terc*^−/−^-*Terc-53* and *Terc*^−/−^-*Terc-53 AS* mice from week 4–16. *n* = 6 male mice per group. (C) The body weight of *Terc*^−/−^ and *Terc*^−/−^-*Terc-53* and *Terc*^−/−^-*Terc-53 AS* mice from month 7–18. *n* = 10 male mice per group. (D) The movement distance of *Terc*^−/−^ and *Terc*^−/−^-*Terc-53* and *Terc*^−/−^-*Terc-53 AS* mice in open field test at 4 months and 18 months of age. *n* = 10 male mice per group. (E) Y maze test of *Terc*^−/−^ and *Terc*^−/−^-*Terc-53* and *Terc*^−/−^-*Terc-53 AS* mice at 4 months and 18 months of age, *n* = 10 (*Terc*^−/−^ male), *n* = 10 (*Terc*^−/−^-*Terc-53* male) and *n* = 7 (*Terc*^−/−^-*Terc-53 AS* male). (F) Schematic of the strategy for conditional expression of *Terc-53* or the RNA with the scrambled sequence (*Scramble*) in 6-month-old G2 *Terc*^−/−^ male mice using AAV, the behavioral tests and LTP analysis. (G) Schematic of Morris water maze test. Mice were analyzed for escape latency during a 5-day training period. On day 6, time spent in the target zone with the platform removed was measured. (H) Escape latency of *Terc*^−/−^; AAV-*Terc-53* (*n* = 10) and *Terc*^−/−^; AAV-*Scramble* (*n* = 11) mice during the 5-day training period. (I) Time spent in target quadrant by *Terc*^−/−^; AAV-*Terc-53* (*n* = 10) and *Terc*^−/−^; AAV-*Scramble* (*n* = 11) mice during probe trail. (J) Representative traveling heatmaps of *Terc*^−/−^; AAV-*Terc-53* and *Terc*^−/−^; AAV-*Scramble* mice during probe trail. (K) Y maze test of *Terc*^−/−^; AAV-*Terc-53* (*n* = 10) and *Terc*^−/−^; AAV-*Scramble* (*n* = 10) mice. (L) LTP recordings in the hippocampal CA1 region of *Terc*^−/−^; AAV-*Terc-53* and *Terc*^−/−^; AAV-*Scramble* mice. *n* = 5 male mice per group. Data are presented as mean ± SEM. ns, no significant difference, *P < 0.05, **P < 0.01.

We were most interested in the effect of normal aging on cognition. Even though adult neurons are terminally differentiated cells, aging neurons have been shown to develop senescence-like phenotypes such as an induction of SA-β-gal activity (Piechota et al., 2016). Additionally, through secretion of inflammatory cytokines, aging neurons could also cause senescence of dividing cells in the brain. Senescence-associated β-galactosidase (SA-β-gal) staining of the hippocampus of the mice showed no difference in the three groups of mice at 4 months of age, but an upregulation of SA-β-gal activity by *Terc-53* overexpression was observed when the mice reached 18 months of age, consistent with the late appearance of aging phenotypes (Fig. S2C and S2D). Hematoxylin and eosin (H&E) staining of the hippocampus of the 18-month-old mice also showed abnormality of neuronal cells in *Terc-53* mice (Fig. S2E). Even though adult human brains contain little telomerase activity, *TERC* has been shown to be expressed in adult brains, with a level comparable to that in fetal brains and the adult livers (Feng et al., 1995). Both *Terc* and *Terc-53* were also detected in N2a cells that are derived from the mouse neural crest (Zheng et al., 2019), suggesting telomerase-independent functions. Since *Terc-53* is derived from *Terc* and mitochondria are involved in its processing and translocation, the levels of proteins involved in mitochondrial biosynthesis and functions and telomerase activity in the hippocampus were examined. No significant difference was observed (Fig. S3), consistent with the previous finding that *Terc-53* functions independent of mitochondria and telomerase (Cheng et al., 2018; Zheng et al., 2019). To further exclude the effects of full-length *Terc* and telomerase, *Terc-53* and *Terc-53 AS* transgenic mice were crossed with *Terc*^−/−^ mice to generate the *Terc*^−/−^-*Terc-53* and *Terc*^−/−^-*Terc-53 AS* mice (Fig. S4). A similar age-dependent effect of *Terc-53* on health in general was observed in *Terc*^−/−^ mice (Fig. 2A–E). The only difference was that *Terc-53* seemed to have an effect on body weight at an earlier age. SA-β-gal staining of the hippocampus of the mice showed an upregulation of SA-β-gal activity by *Terc-53* overexpression at an early age of 4 months (Fig. S5A), which might explain the earlier onset of body weight loss and worse phenotypes at later stages of life. The percentage of β-gal-positive cells increased in all three groups of mice as they aged (Fig. S5B). The changes of other aging markers of the brain including p53, Bdnf (brain-derived neurotrophic factor) and GluA1 (an AMPA receptor subunit) (Lu et al., 2004) also appeared as early as 4 months of age (Fig. S5C and S5D). That the behavioral defects appeared later than the molecular changes in the brain might be explained by the functional network redundancy of the brain to withstand damages (Sadiq et al., 2021). For example, brain changes could be detected 20 years before the Alzheimer’s symptoms (Reiman et al., 2012).

Since the transgenic mice were generated with random insertion, there was a small possibility that these effects might be due to the site of insertion. To rule out this possibility, *Terc-53* or the RNA with the scrambled sequence (*Scramble*) was conditionally expressed in the hippocampus of 6-month-old G2 *Terc*^−/−^ male mice using AAV delivery of the RNA expression constructs (Fig. 2F). Since neuron abnormality was observed in the 18-month-old *Terc-53* transgenic mice but there was no neuron-specific RNA promoter available, we decided to use the neuron-specific human synapsin 1 gene (Hsyn) promoter instead. *Terc*^−/−^ mice with *Terc-53* conditionally expressed in the neuron of the hippocampus showed a significant decline of learning ability (based on Morris water maze test) and short-term memory (Y-maze test), compared to the *Scramble* RNA-expressing mice (Fig. 2G–K). The induction of long-term potentiation was also significantly reduced in the hippocampus of *Terc-53*-expressing mice (Fig. 2L). Taken together, these data support a role of *Terc-53* in aging of mammals.

To understand how *Terc-53* functions in aging of the brain, we first examined its expression in different brain regions. Northern blot showed that the RNA is expressed in the cortex, the hippocampus and the brainstem of the 3-month-old and 18-month-old wild-type mice (Fig. S6A and S6B). The cerebellum has very little of the RNA even though the full-length *Terc* was expressed at a similar level in the four regions (Fig. S6A and S6B). The levels increase in the cortex and the hippocampus, but decrease in the brainstem, as the mice age (Fig. S6A and S6B). We have shown previously that the overall *Terc-53* levels increase in the mouse brain as the mice age, reach a peak at 10 months of age, and then drop close to 4-month-old levels at 18 months of age, suggesting compensatory feedback in the later stage of aging process (Zheng et al., 2019). This compensatory feedback could be brain region-specific or stronger in the brainstem than the other regions. The exact feedback mechanism remains to be elucidated. Primary cells were also isolated from the brain of the 3-month-old wild-type mice and *Terc-53* expression levels in different brain cells were again examined by Northern blot. The RNA was detected in neurons as expected, but surprisingly, it was not detected in astrocytes or microglia, even though the full-length *Terc* was expressed at the same level in all three types of cells (Fig. S6C). Interestingly, exogenous expression of *Terc-53* in the hippocampus of 6-month-old *Terc*^−/−^-*Terc-53* mice using a non-specific U6 promoter was also only detected in neurons, suggesting a post-transcriptional regulation (Fig. S6D and S6E). Indeed, *Terc-53* became detectable in astrocytes and microglia isolated from *Terc*^−/−^-*Terc-53* mice after the cells were treated with RNA inhibitors (Fig. S6F). Treatment of the cells with Actinomycin D (a transcription inhibitor) led to a reduction of total RNA levels, while treatment with RNA inhibitors reversed the reduction, suggesting that it was the RNA degradation that was inhibited by the RNase inhibitors (Fig. S6F). Therefore, *Terc-53* is regulated by selective degradation in astrocytes and microglia, but not the neurons.

### *Terc-53* interacts with Hmmr and accelerates its degradation

**Figure 3.**
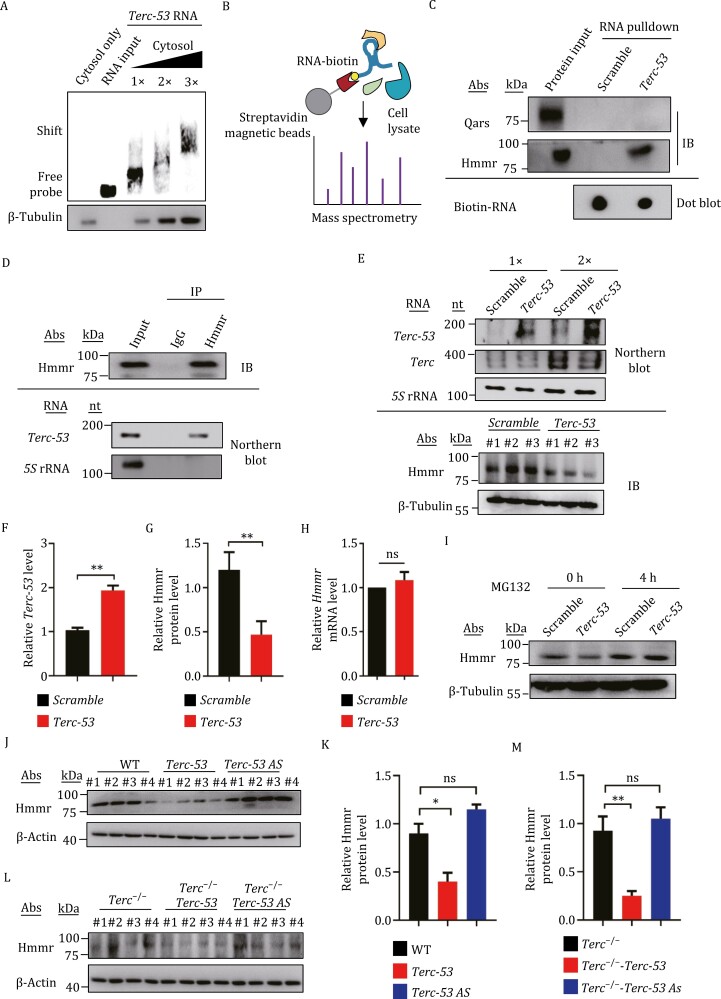
***Terc-53* interacts with Hmmr and accelerates its degradation.** (A) Gel shift assay of biotin-labeled *Terc-53* RNA with the cytosolic lysate of N2a cells. The first lane is the cytosol without the biotin-labeled RNA and the second lane is the biotin-labeled RNA without the cytosol. (B) Schematic of the RNA-protein pulldown from the cytosolic lysate of N2a cells and Mass spectrometry analysis of the purified proteins. Biotin-labeled *Terc-53* was used as the bait and Streptavidin beads were used to pulldown the RNA-protein complexes. (C) Biotinylated *Terc-53* or the RNA with the scrambled sequence (*Scramble*) was incubated with N2a cytosolic lysates for the RNA pulldown assay, followed by immunoblotting of the indicated proteins. The bottom panel is a dot blot of the biotin-labeled RNAs. (D) IgG and Hmmr antibody were used to immunoprecipitate potential interacting RNAs. The purified RNAs were examined by Northern blot using the biotin-labeled RNA probes as indicated. (E) Northern blot analysis of the expression of *Terc-53* and full-length *Terc* in *Terc-53* overexpressing N2a cells (*Terc-53*) or the cells expressing the RNA with the scrambled sequence (*Scramble*)*. 5S* RNA was used as the loading control. Bottom panels are an immunoblot of Hmmr and that of β-tubulin as a loading control. (F) Quantification of *Terc-53* levels in panel (E). (G) Quantification of Hmmr protein levels in panel (E). (H) *Hmmr* mRNA levels in *Terc-53*-overexpressing N2a cells (*Terc-53*) or the cells expressing the *Scramble* RNA (*Scramble*), analyzed by RT-qPCR. (I) Immunoblotting of Hmmr protein levels in *Terc-53*-overexpressing N2a cells (*Terc-53*) or the cells expressing the *Scramble* RNA (*Scramble*), before and after MG132 (a proteasome inhibitor) treatment. β-Tubulin was used as a loading control. (J) Immunoblotting of Hmmr protein levels in the hippocampus of WT, *Terc-53* and *Terc-53 AS* male mice at 18 months of age. β-Actin was used as a loading control. (K) Quantification of Hmmr protein levels in panel (J). (L) Immunoblotting of of Hmmr protein levels in the hippocampus of *Terc*^−/−^, *Terc*^−/−^-*Terc-53,* and *Terc*^−/−^-*Terc-53 AS* male mice at 18 months of age. β-Actin was used as a loading control. (M) Quantification of Hmmr protein levels in panel (L). Data are mean ± SD from three experiments. ns, not significant; *P < 0.05; **P < 0.01; ***P < 0.001.

Since primary cells are expensive to acquire and difficult to maintain, we decided to use N2a cells (a mouse neural crest-derived cell line) to look for *Terc-53*’s targets and downstream effectors and verify them later in primary neurons and *in vivo*. Cellular processes that are involved in aging of the brain (Dionisio et al., 2021; Fleming et al., 2022) were examined in *Terc-53*-overexpressing or -knockdown N2a cells. Changes of the RNA levels in either direction did not induce autophagy or apoptosis-like D-gal did (Fig. S7), suggesting a different and milder regulatory role, which is consistent with the slow onset of the aging effect in mice. Gel shift assay of biotin-labeled *Terc-53* with the cytosolic lysate of N2a cells showed a few higher bands (Fig. 3A), indicating potential binding partners in the lysates. To identify the potential *Terc-53* binding proteins, biotin-labeled *Terc-53* was incubated with N2a cytosolic lysate, and streptavidin beads were used to pulldown the RNA-protein complexes (Fig. 3B). Mass spectrometry of the eluate identified Hmmr as one of the top candidates (Fig. S8A; Tables S2 and S3). Pulldown of the protein by *Terc-53* was verified with immunoblotting of the eluates with a commercial Hmmr antibody (Fig. 3C).

Most of the study on Hmmr was focused on its association with cancer (Heldin et al., 2013; Mateo et al., 2022). The exact role it plays in oncogenesis, however, is unclear. The outcome of its effect might be regulated in context-specific manner, depending partially on the molecular weight of hyaluronan (Rahman et al., 2020; Tian et al., 2013; Zhang et al., 2023a). What piqued our interest was the report that Hmmr might contribute to the longevity of naked mole-rat *Heterocephalus glaber* (Keane et al., 2014). A quick search and analysis of an existing Alzheimer’s disease (AD) database [Gene Expression Omnibus (GEO) accession number GSE118553] showed that *HMMR* mRNA levels are downregulated in the Cortex of the AD patients while relatively unchanged in the cerebellum (Fig. S8B and S8C), which reminded us of the expression pattern of *Terc-53* in these two regions of the brain (Fig. S6A and S6B). In addition, immunoblotting examination of Hmmr in the hippocampus of 4-month-old and 18-month-old mice showed that the protein levels were significantly reduced in the 18-month-old (Fig. S8D and S8E), suggesting its involvement in normal aging. The aging-related expression patterns of Hmmr in other tissues were also examined. A downregulation of the protein levels was also observed in the lungs and the liver, while an upregulation was observed in the spleen (Fig. S8F and S8G). The protein levels did no change in the heart as the mice aged (Fig. S8F and S8G). These results suggest that Hmmr’s involvement in aging may be tissue specific. Analyzing the existing RNAseq database of young and aged human brains also revealed a downregulation of *HMMR* expression levels as they age (Fig. S8H). In APP-overexpressing N2a-APP695 cells, Hmmr protein levels were significantly reduced (Fig. S8I and S8J). Similar changes were also observed in the hippocampus of 5× FAD mice at 6 months of age with immunoblotting of the hippocampus lysate or immunofluorescent staining of the brain section (Fig. S8K–N). The changes of Hmmr protein levels are similar to the changes of *Terc-53* RNA levels *in vitro* and *in vivo* in these disease models, only in the opposite direction (Fig. S1), suggesting Hmmr as an effector of *Terc-53* in aging of the brain.

The physical interaction and functional interaction between *Terc-53* and Hmmr were further characterized. The physical interaction between *Terc-53* and Hmmr was again verified by immuno-coprecipitation. Northern blot of the sample immunoprecipitated with Hmmr antibody identified *Terc-53*, but not *5S* rRNA (Fig. 3D). The interaction is specific between Hmmr and *Terc-53*, as the full-length *Terc* did not pulldown Hmmr even though the protein component of telomerase Tert was copurified with it (Fig. S9A). The physical interaction between Hmmr and *Terc-53* was further verified with a gel shift assay of the purified protein and biotin-labeled RNA (Fig. S9B and S9C).

The sites of interaction on *Terc-53* and Hmmr were also extensively mapped out (Figs. S10 and S11). Based on the predicted secondary structure of *Terc-53*, different fragments of *Terc-53* that harbor one to three of the three stem-loops were generated (Fig. S10A). The biotin-labeled RNA fragments were used to pulldown purified Hmmr. All the fragments that include nt 102–130 retained the full capacity to interact with Hmmr (Fig. S10B). Mutation of the most conserved seven nucleotides (nt 117–123) that form the right side of the bulge at the bottom of the smallest stem-loop (*Terc-53* mut) completely abolished the interaction (Fig. S10C–E). Different fragments of Hmmr with Myc tag was also generated to immunoprecipitate *Terc-53*. Since no functional domains have been reported, truncations of two halves and four quarters were generated (Fig. S11A). All the constructs that contain the C-terminus (aa 596–794) were able to pulldown *Terc-53* (Fig. S11).

The functional consequence of *Terc-53* and Hmmr interaction was manifested in Hmmr protein levels. Overexpression of *Terc-53* led to a significant reduction of Hmmr protein levels in N2a cells (Fig. 3E–G). Knockdown of *Terc-53* with the antisense RNA led to the opposite effect (Fig. S12A). The effect is dosage dependent and requires the physical interaction between *Terc-53* and Hmmr, as the mutation that abolishes the interaction removed the effect (Fig. S12B and S12C). The reduction was not due to regulation on the transcriptional level or at the protein synthesis step (Figs. 2H and S12D), but to a change of protein degradation rate by proteasomes. Treating the cells with MG132, a proteasome inhibitor, removed most of the effect of *Terc-53* (Fig. 3I). In contrast, inhibiting autophagy with chloroquine had no such result (Fig. S12E).

The negative effect of *Terc-53* on Hmmr protein stability was also observed in the transgenic mice. Hmmr protein levels were significantly lower in the hippocampus of *Terc-53* mice compared to the wild-type control (Fig. 3J and 3K). A small increase of the protein levels was observed in *Terc-53 AS* mice, but bears no statistical significance, which might explain the lack of phenotypes in these mice, as the effect is most likely dosage dependent (Fig. 3J and 3K). Similar effect of *Terc-53* was also observed in *Terc*^−/−^-*Terc-53* mice, without the influence of endogenous *Terc* or telomerase (Fig. 3L–M).

Since poly-ubiquitination is generally required for proteasome degradation of the protein substrates, we analyzed Hmmr amino acid sequence using PhosphoSitePlus, and found five potential ubiquitination sites (Fig. S13A). Overexpression of *Terc-53* led to an upregulation of the ubiquitination levels of Hmmr, which is also dependent on the Hmmr-binding capacity of the RNA, as overexpression of the *Terc-53* mutant (*Terc-53* mut) that does not bind Hmmr had no such effect (Fig. S13B). Immunoblotting of the purified Hmmr with a monoclonal antibody that specifically recognizes K48-linked or K63-linked polyubiquitin chains showed that *Terc-53* regulates K48-linked polyubiquitination of Hmmr but not K63-linked polyubiquitination (Fig. S13C–E).

### *Terc-53* functions as a scaffold for Trim25-mediated K48-linked ubiquitination of Hmmr

**Figure 4.**
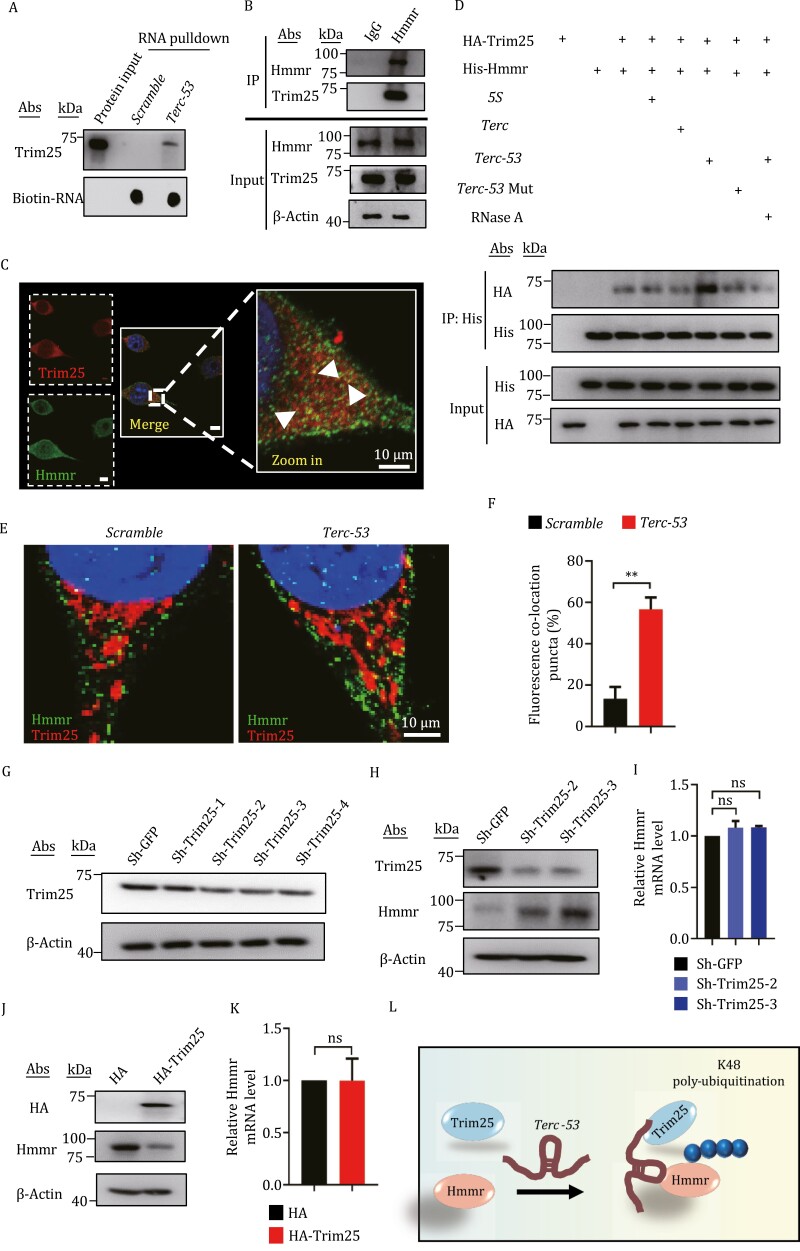
***Terc-53* functions as a scaffold for Trim25-mediated K48-linked ubiquitination of Hmmr.** (A) Biotinylated *Terc-53* or the RNA with the scrambled sequence (*Scramble*) was incubated with N2a cytosolic lysates for the RNA pulldown assay, followed by immunoblotting of Trim25. The bottom panel is a dot blot of the biotin-labeled RNAs. (B) Co-immunoprecipitation of Trim25 with Hmmr. Hmmr antibody was used to immunoprecipitate potential binding proteins. The purified protein complexes were examined by immunoblotting with Trim25 antibody. (C) Immunofluorescence staining of Hmmr (Alexfluo 488) and Trim25 (Alexfluo 594) in N2a cells. White arrows indicate the sites of co-localization. (D) *In vitro* interacting assay of Hmmr, Trim25 and *Terc-53*. His-tagged Hmmr and HA-tagged Trim25 were mixed with *in vitro* transcribed *Terc-53*, *5S* rRNA, full-length *Terc* or the *Ter-53* mutant (*Terc-53* Mut) that does not bind Hmmr (shown in Fig. S8C–E). The complexes were immunoprecipitated with His antibody, and the levels of co-purified Trim25 was examined with HA antibody. One sample with *Terc-53* and the two proteins was also treated with RNase A before the immunoprecipitation. (E) Immunofluorescence staining of Hmmr (Alexfluo 488) and Trim25 (Alexfluo 594) in *Terc-53*-overexpressing (*Terc-53*) or the *Scramble* RNA-expressing (*Scramble*) N2a cells. (F) Quantification of the colocalization puncta in panel (E). (G) Immunoblotting examination of Trim25 protein levels in N2a cells with different shRNA constructs for Trim25 knockdown. A shRNA construct for GFP was used as a negative control. β-Actin was used as a loading control. (H) Immunoblotting examination of Hmmr protein levels in Trim25-knockdown or the control (sh-GFP) N2a cells. β-Actin was used as a loading control. (I) *Hmmr* mRNA levels in Trim25-knockdown or the control (sh-GFP) N2a cells. (J) Immunoblotting examination of Hmmr protein levels in N2a cells with (HA-Trim25) or without (HA) Trim25 overexpression. (K) *Hmmr* mRNA levels in N2a cells with (HA-Trim25) or without (HA) Trim25 overexpression. (L) Model of *Terc-53* functioning as a molecular scaffold to enhance Trim25-mediated Hmmr K48-linked polyubiquitination. The small blue circle indicates ubiquitin molecules. Data are mean ± SD from three experiments. ns, not significant; *P < 0.05; **P < 0.01; ***P < 0.001.

We searched the Mass spectrometry data of *Terc-53*-interacting proteins and found an E3 ligase Trim25. Trim25 is an RNA-binding protein that prefers RNA with high GC content (Choudhury et al., 2017). Coincidently, *Terc-53* happens to be an RNA with very high GC content (79.6%) (Fig. S13F). The interaction between *Terc-53* and Trim25 was confirmed with RNA pulldown and immunoblotting with a Trim25 antibody (Fig. 4A). The interaction between Hmmr and Trim25 was confirmed with immuno-coprecipitation using the Hmmr antibody and immunoblotting using the Trim25 antibody (Fig. 4B). Colocalization of the two proteins was also observed by immunofluorescent microscopy using the two antibodies. The interaction among *Terc-53*, Hmmr, and Trim25 was examined using an *in vitro* binding assay with *in vitro*-synthesized *Terc-53* and purified His-tagged Hmmr and HA-tagged Trim25 (Figs. 4D, S9B and S13G). Hmmr interacted with Trim25 without *Terc-53*, but addition of *Terc-53* improved the interaction (Fig. 4D). *Terc-53* mutant (*Terc-53* mut) that does not bind Hmmr had no such positive effect on interaction between Hmmr and Trim25 (Fig. 4D). Treating the mixture of *Terc-53*, Hmmr and Trim25 with RNase A also removed the effect (Fig. 4D), suggesting that *Terc-53* functions as a scaffold facilitating the interacting between Hmmr and Trim25. This interaction-facilitating effect was also observed with immunofluorescent microscopy. More colocalization puncta between Hmmr and Trim25 were observed in *Terc-53*-overexpressing N2a cells compared to the vector-control cells (Fig. 4E and 4F). Knockdown of Trim25 with shRNAs led to an increase of Hmmr levels in N2a cells, while overexpression had the opposite effect (Fig. 4G–K). Again, the effects were post-transcriptional, as the mRNA levels of *Hmmr* were unchanged (Fig. 4I–K). The effect of Trim25 overexpression on Hmmr protein levels was partially removed by expression of *Terc-53* antisense RNA (Fig. S13H and S13I), further proving the functional interaction of the three.

The sites of ubiquitination in Hmmr was identified by immunoprecipitation of Hmmr from HEK293T cells overexpressing mouse Hmmr, Trim25 and ubiquitin, and Mass spectrometry analysis of the purified samples. HEK293T cells instead of N2a were used because co-transfection of three constructs failed with N2a cells. Co-transfection ensured that most of mouse Hmmr was ubiquitinated by mouse Trim25. Two ubiquitination sites were identified: one at K265 and the other at K736, each on an α-helix of coiled coil structure predicted by Alphafold2 (Fig. S14A). Mutation of the two Lysines on both sites to Arginines stabilized Hmmr, rendering it insensitive to *Terc-53* regulation (Fig. S14B and S14C).

The interaction between *Terc-53*, Hmmr and Trim25 was also observed in primary neurons. Biotin-labeled *Terc-53* coprecipitated Hmmr and Trim25 from the lysate of of 16-day DIV (days *in vitro*) *Terc*^*−/−*^ primary neuron cells (Fig. S15A). *Terc-53* RNA FISH, and Hmmr and Trim25 immunostaining in *Terc*^−/−^-*Terc-53* primary neuron showed colocalization of the three (Fig. S15B).

Together, these data suggest that *Terc-53* functions as a scaffold bringing together Hmmr and Trim25 for the K48-linked polyubiquitination of Hmmr at K265 and K736, which leads to degradation of Hmmr by proteasomes (Fig. 4L).

### *Terc-53* accelerates brain aging by promoting astrocyte-mediated inflammation

**Figure 5.**
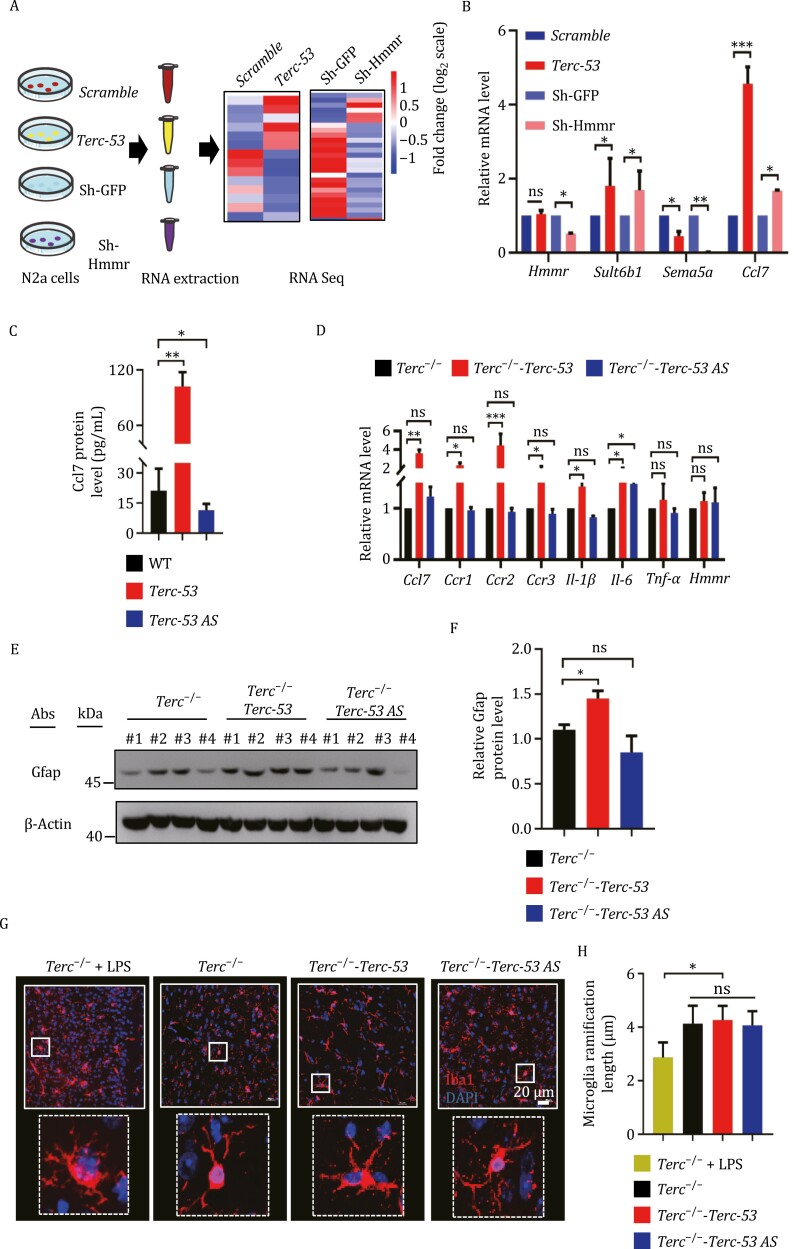
***Terc-53* accelerates brain aging by promoting astrocyte-mediated inflammation.** (A) Schematic of RNA-seq sample preparation and analysis. Total RNAs were isolated from N2a cell lines as indicated. Heatmaps of differentially expressed (FDR < 0.05) genes are shown on the right. Genes with expression affected similarly by *Terc-53* overexpression and Hmmr knockdown were chosen for further verification. (B) RT-qPCR examination of co-regulated genes in the indicated cell lines. (C) ELISA measurement of Ccl7 protein levels in the hippocampus of 18-month-old WT, *Terc-53* and *Terc-53 AS* male mice (*n* = 3). (D) RT-qPCR analysis of the levels of the mRNAs as indicated in the hippocampus of *Terc*^−/−^, *Terc*^−/−^-*Terc-53* and *Terc*^−/−^-*Terc-53 AS* male mice at 18 months of age (*n* = 4). (E) Immunoblotting of Gfap protein (an astrocyte marker) in the hippocampus of *Terc*^−/−^, *Terc*^−/−^-*Terc-53* and *Terc*^−/−^-*Terc-53 AS* male mice at 18 months of age. (F) Quantification of the relative Gfap protein levels in panel (E). (G) Immuno-staining of Iba1 (a microglia marker) in the hippocampus of *Terc*^−/−^, *Terc*^−/−^-*Terc-53* and *Terc*^−/−^-*Terc-53 AS* male mice at 18 months of age. The image of *Terc*^−/−^ mice with 2 mg/kg LPS intraperitoneally injected four days before the dissection was used as a microglia activation positive control. (H) Quantification of the ramification length in panel (G) (*n* = 3 male mice per group). Data are mean ± SD from three experiments. ns, not significant; *P < 0.05; **P < 0.01; ***P < 0.001.

To understand how *Terc-53* and Hmmr regulate aging of the brain, we analyzed the transcriptomes of *Terc-53*-overexpressing N2a cells and Hmmr-knockdown cells (Fig. 5A). Knockdown of Hmmr using shRNAs had no effect on Trim25 protein levels or *Terc-53* RNA levels (Fig. S16A and S16B), suggesting it at the downstream of the two. 340 genes showed altered expression in *Terc-53*-overexpressing cells (> 2-fold difference to *Scramble* control, FDR-corrected *P* < 0.01) (Fig. S16C), while 464 genes showed altered expression in Hmmr-knockdown cells (> 2-fold difference to Sh-GFP control, FDR-corrected *P* < 0.01) (Fig. S16D). A total of 62 regulated genes were identified as overlapping set of targets between *Terc-53-* and Hmmr-regulated transcriptomes (Fig. S16E; Tables S4 and S5). A positive correlation between *Terc-53* overexpression and Hmmr knockdown was also revealed by the analysis (Fig. S16F). Transcriptional coregulation of these genes were verified by RT-qPCR (Fig. 5B). The expression of one with no established function in the brain *Sult6b1*, one with association to Autism *Sema5a* (Ito et al., 2017), and one with well-established role in the brain *Ccl7* (Ke et al., 2016; Wang et al., 2018) was examined (Fig. 5B). Gene Ontology (GO) pathway analysis of these co-regulated genes enriched Il-17 signaling pathway and cytokine-cytokine receptor interaction among the most significant categories (Fig. S16G).

The GO enriched pathways suggest that neuroinflammation might be involved in *Terc-53-* and Hmmr-mediated brain aging. One of the co-regulated genes *Ccl7* encodes a proinflammatory cytokine (Jorda et al., 2021; Ke et al., 2016; Sogorb-Esteve et al., 2021; Wang et al., 2018). ELISA measurement of the protein levels showed that it was upregulated in the hippocampus of *Terc-53* mice, while slightly downregulated in *Terc-53 AS* mice at 18 months of age (Fig. 5C). RT-qPCR showed an upregulation of *Ccl7* mRNA levels in *Terc*^−/−^-*Terc-53* mice but no difference was observed in *Terc*^−/−^-*Terc-53 AS* mice (Fig. 5D). Similar changes were observed in the mRNA levels of its receptor *Ccr1-3* (Jorda et al., 2021) and those of another cytokines *Il-1β* that has been shown to be involved in the induction of learning and memory deficits (Cunningham and Sanderson, 2008; Sabouri et al., 2020) (Fig. 5D). Expression of *Terc-53 AS* led to upregulation of *Il-6*, suggesting some off-target effect, which might also explain why a positive effect on aging animals was not observed in the *Terc-53* knockdown mice (*Terc-53 AS* mice, wild-type background). The expression levels of *Il-1β*, *Il-6*, and *Tnf-α* were also examined in the 4-month-old mice. No differences were observed in the three groups of mice on the wild-type background (Fig. S17A). Both *Il-1β* and *Il-6* levels were upregulated in the hippocampus of *Terc*^−/−^-*Terc-53* mice even at the young age (Fig. S17B). The protein levels of another cytokine Il-17, which is closely related to neuroinflammation and cognition decline were also upregulated in the hippocampus of *Terc*^−/−^-*Terc-53* mice at both ages, while no difference of NF-κB p65 or phospho-NF-κB p65 (Ser536) protein levels was observed (Fig. S17C–G). Immunoblotting the hippocampus lysates of 18-month-old *Terc*^−/−^, *Terc*^−/−^-*Terc-53,* and *Terc*^−/−^-*Terc-53 AS* mice revealed an upregulation of Gfap protein levels in *Terc*^−/−^-*Terc-53* mice, an indication of astrocyte activation (Colombo and Farina, 2016) (Fig. 5E and 5F). In contrast, immunostaining of microglia in the hippocampus of the three groups of mice did not show a change of ramification patterns, unlike the treatment with LPS that caused a shortening of the ramifications (Wang et al., 2018), suggesting that microglia are not involved in *Terc-53*-mediated neuroinflammation (Fig. 5G and 5H).

**Figure 6.**
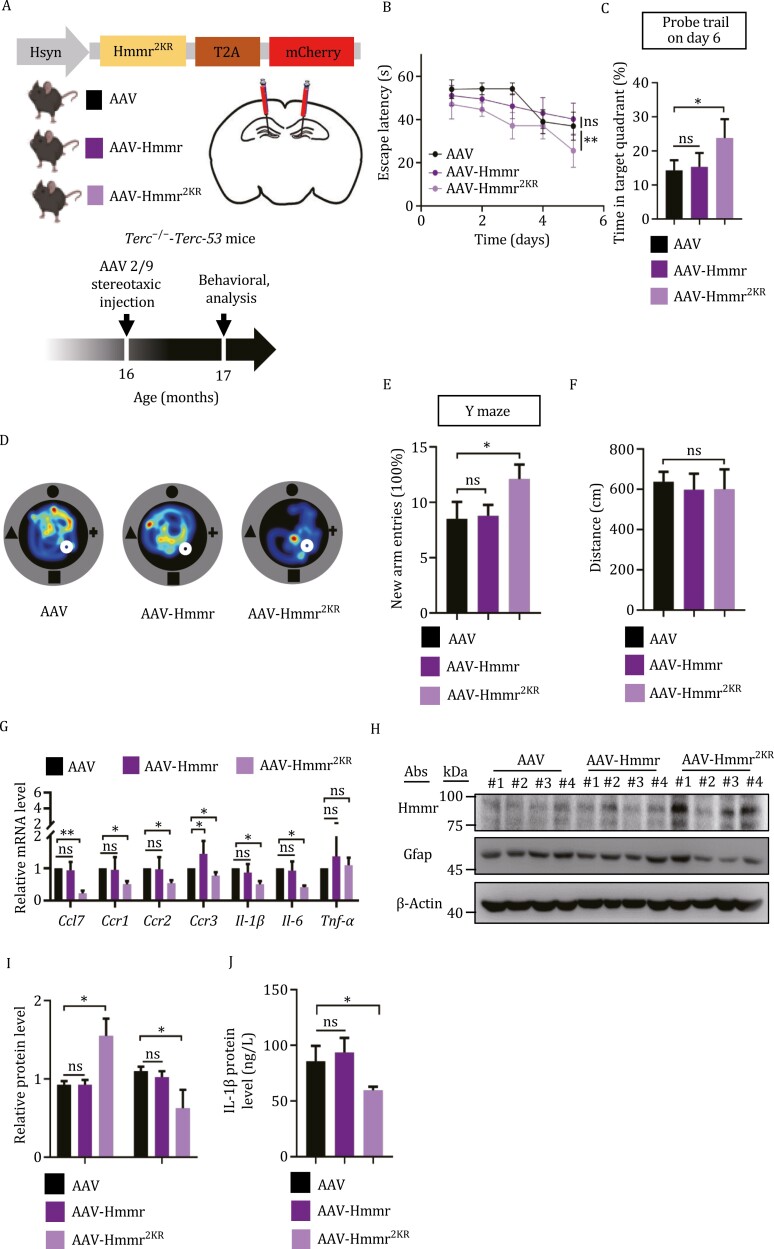
**Hmmr^2KR^ alleviates *Terc-53*-mediated cognition-decline and inflammation.** (A) Schematic of the strategy for conditional overexpression of Hmmr or the ubiquitination-deficient mutant Hmmr^2KR^ (shown in Fig. S12) in 16-month-old *Terc*^−/−^-*Terc-53* male mice, and the behavioral tests. (B) Escape latency of *Terc*^−/−^-*Terc-53*;AAV, *Terc*^−/−^-*Terc-53*;AAV-Hmmr and *Terc*^−/−^-*Terc-53*;AAV-Hmmr^2KR^ mice in Morris water maze during the 5-day training period. *n* = 9 male mice per group. (C) Time spent in target quadrant by *Terc*^−/−^-*Terc-53*;AAV, *Terc*^−/−^-*Terc-53*;AAV-Hmmr and *Terc*^−/−^-*Terc-53*;AAV-Hmmr^2KR^ mice in Morris water maze during probe trail. *n* = 9 male mice per group. (D) Representative traveling heatmaps of the three groups of mice during probe trail. (E) Y maze test of the three groups of mice. *n* = 9 male mice per group. (F) The movement distance of the three groups of mice in open field test. *n* = 8 male mice per group. (G) RT-qPCR analysis of the levels of the mRNAs as indicated in the hippocampus of the three groups of male mice (*n* = 4). (H) Immunoblotting of Hmmr and Gfap proteins in the hippocampus of the three groups of male mice. (I) Quantification of the relative protein levels in panel (H). (J) ELISA measurement of Il-1β protein levels in the hippocampus of the three groups of mice (*n* = 4). Data are presented as Mean ± SEM (B–G) and Mean ± SD (I–J). ns, no significance, *P < 0.05, **P < 0.01.

To establish whether there is a causal relationship between *Terc-53*-mediated Hmmr degradation, neuroinflammation, and brain aging, we tried to restore Hmmr protein levels in 16-month-old *Terc*^−/−^-*Terc-53* mice using AAV delivery of the protein-expressing constructs and examine whether it would reverse the aging phenotypes (Fig. 6A). Two constructs were used: one expressing the wild-type Hmmr, and the other expressing the protein with the ubiquitination sites mutated (Hmmr^2KR^). Microinjection of AAV carrying the ubiquitination-deficient mutant Hmmr^2KR^ construct (AAV-Hmmr^2KR^) but not the wild-type construct (AAV-Hmmr) or the empty vector (AAV) significantly improved the learning ability (based on Morris water maze test), and short-term memory (Y-maze test) of *Terc*^−/−^-*Terc-53* mice without affecting their moving ability (Fig. 6B–F). Neuroinflammation markers were examined in the hippocampus of the three groups of mice. AAV-Hmmr^2KR^ treatment led to a reduction of the expression levels of the proinflammatory cytokine *Ccl7* (Fig. 6G). Similar changes were observed in the mRNA levels of its receptor *Ccr1-3* and those of two other cytokines *Il-1β* and *Il-6* (Fig. 6G). A reduction of Gfap and Il-1β protein levels were also observed in the AAV-Hmmr^2KR^-treated hippocampus (Fig. 6H–J). Unlike AAV-Hmmr^2KR^ treatment, AAV-Hmmr did not increase Hmmr protein levels, a likely reason that it did not mitigate neuroinflammation and reverse cognition decline (Fig. 6H and 6I).

Overall, our results demonstrate that *Terc-53* regulates organismal aging through mediating the stability of hyaluronan receptor Hmmr and neuroinflammation in the brain. These findings underscore the complexity of aging in mammals and the importance of noncoding RNA and proteins of later evolutionary emergence in the process.

## Discussion

As smaller RNAs derived from the RNA component of telomerase *TERC*, *TERC-53* localizes mainly in the cytosol. The physiological functions of the RNA and the molecular mechanisms were largely unknown. Our study has uncovered a telomerase-independent role of *Terc-53* in organismal aging of mice. In the mouse brain, *Terc-53* is expressed mostly in neurons, where it functions as a scaffold for the interaction between Hmmr and Trim25, accelerating Hmmr’s ubiquitination by Trim25 and subsequence degradation, leading to neuroinflammation and cognition decline.

HMMR is one of the main receptors of hyaluronan. It has two hyaluronan binding domains that contain two basic amino acids spaced seven amino acids apart (Yang et al., 1994). HMMR’s association with cancer is well established. Its expression is upregulated in various types of cancer (Heldin et al., 2013). Overexpression in fibroblasts has been shown to be transforming and knockdown leads to resistance of H-ras transformation (Hall et al., 1995). Recent discoveries have also linked HMMR to neural development. Mutation or loss of Hmmr in vertebrate animal models induces enlarged ventricles and megalencephaly (Connell et al., 2017; Li et al., 2017). Our results show that Hmmr plays a protective role against aging of the brain. These findings suggest that the same protein could have different roles in different tissues or in the same tissue at different stages, which probably depend on the differentially-expressed binding partners that it interacts with.

Similarly, multiple molecular mechanisms are likely at work for *Terc-53*’s involvement in organismal aging. In the fast-dividing HEK293 cells, *TERC-53* has been shown to interact with GAPDH and promotes cellular senescence when overexpressed (Zheng et al., 2019). In neurons, it accelerates Hmmr’s degradation, causing neuroinflammation. Both mechanisms have been shown to contribute to organismal aging. The proportion each mechanism contributes to the aging of a certain organ, therefore, also depends on the cell types and the expression profiles of the interacting partners in the organ. In this study, we focused mainly on the cognition side of brain aging. We have shown that *Terc-53* is specifically expressed in neurons. The Hmmr-mediated mechanism, therefore, becomes the main driving factor.

The effects of *Terc-53* overexpression on aging take time to manifest. Unlike most fast-aging mouse models that show developmental problems, mental retardation or an increase of the incidence of tumors (Barlow et al., 1996; Chang et al., 2004; Fatkin et al., 1999; Varela et al., 2005), our model is a rare one that recapitulates most features of normal aging without all these maladies. Hmmr functioning as the main downstream effector of *Terc-53* in mouse brain also aligns with a recent finding showing that increased hyaluronan by Has2 from naked mole-rat improves healthspan in mice (Zhang et al., 2023a). Naked mole-rats have a maximum lifespan of over 40 years, almost twenty times the lifespan of most mice. Expression of naked role-rat Has2, however, has very limited effect on mouse lifespan, despite its effects on healthspan (Zhang et al., 2023a). Maybe Hmmr is a missing link on the lifespan story of hyaluronan. We have shown that Hmmr levels drop as animals or humans age. Co-expressing the ligand (hyaluronan) and the receptor might lead to a better effect on both health and lifespan, which is still to be verified with animal models.

Our findings also have a couple of implications for the theories of aging. Telomerase activity is normally limited to stem, germ and the majority of cancer cells, but *TERC* is expressed in a majority of somatic cells where it is expressed constitutively (Feng et al., 1995). *TERC-53* adds a new layer to the regulation. It has become an essential part of *TERC*’s story on aging. That neuroinflammation plays an important role in *TERC-53*-mediated aging of the brain is a new piece of evidence supporting the inflammation theory of aging. Inflammation as a driving factor of normal aging seems more consistent with the slowly-manifested effects. High levels of age-associated pro-inflammatory markers have been detected in the majority of older individuals, even in the absence of risk factors and clinically active diseases (Ferrucci and Fabbri, 2018). In our mouse models, higher levels of inflammation were observed in the brain of 18-month-old mice than that of 4-month-old. Overexpression of *Terc-53* exacerbates the effect. Deletion of *Terc*, which itself upregulates inflammation (Yang et al., 2022), accelerates the deterioration. Since hyaluronan and HMMR appear late in evolution (Stern, 2017) and both *C*. *elegans* and Drosophila lack *TERC*, it would be also interesting to explore this evolutionarily non-conserved side of aging.

## Methods

### Cell culture and transfection

HEK293T cells, N2a cells and N2a-App695 cells were cultured in Dulbecco’s modified Eagle’s medium (DMEM) containing 4.5 g/L glucose, 1 mmol/L sodium pyruvate and 4 mmol/L L-glutamine, supplemented with 10% fetal bovine serum. Cells were grown in 5% CO_2_ at 37°C. All cell lines used in this study were mycoplasma negative.

Primary cortical neurons were isolated from cerebral cortices of WT, *Terc*^−/−^ or *Terc*^−/−^-*Terc-53* mouse pups (postnatal day 1). Tissues were removed and put into ice-cold D-Hank’s balanced salt solution containing 10 mmol/L HEPES, chopped into small strips, and digested by papain (Solarbio, Cat#G8430, 1 mg/mL) for 20 min at 37°C. DMEM with 10% heat-inactivated fetal bovine serum was added to terminate the digestion reaction. After trituration, cells were precipitated by centrifugation at 1000 ×*g* and re-suspended in NeuroBasal medium (Gibco, Cat# 21103049) containing 2% B27 (Gibco, Cat# A3582801), and 1% penicillin/streptomycin.

Primary astrocytes were isolated from P1 mice as previously described (Zhang et al., 2023b). The isolation process was the same as that of neurons, except that the primary astrocytes were cultured in DMEM/F12 with 100 μg/mL streptomycin, 100 U/mL penicillin, and 10% fetal bovine serum.

Primary microglia were isolated from P0 mice with the same protocol and cultured in DMEM with GMCSF, 100 μg/mL streptomycin, and 100 U/mL penicillin.

For actinomycin D (MCE, Cat# HY-17559) and RNase inhibitor (Thermo, Cat# EO0382) treatment, 10 μg/mL actinomycin D and 100 U/mL RNase inhibitors were used to treat primary *Terc*^−/−^-*Terc-53* neuron, astrocyte and microglia for 2 h and 1 h respectively.

Transient transfection of plasmids and shRNAs were performed using Lipofectamine 3000 (Invitrogen, Cat# 11668019) reagent according to the manufacturer’s instructions. To generate stable cell lines, HEK293T cells were transfected with VSVg and Hit60 packaging vectors and the plasmid of interest using TurboFect (Thermo). Harvested retroviruses were used to infect the cells of interest, followed by selection with 5 μg/mL puromycin.

### Cell senescence staining

Commercial kit (Sigma, Cat# CS0030) was used to examine the senescence of the cells. Mouse brain slices were fixed with 2% PFA for 15 min at room temperature. After fixation, the brain slices were washed 3 times with PBS and then incubated overnight at 37°C without CO_2_ in freshly prepared staining buffer. 10–20 random visual fields were captured for all samples. Images were quantified by manual counting of SA-β-Gal positive cells by a blinded assessor.

### Enzyme-linked immunosorbent assay (ELISA)

Il-1β, Il-17, and Ccl7 expression levels in mouse brains were measured using mouse Il-1β, Il-17, and Ccl7 ELISA Kits (Neobioscience, mouse Il-1β ELISA kit, EMC001b, Neobioscience, mouse Il-17 ELISA kit, EMC008 and Solarbio mouse-Ccl7 ELISA Kit, SEKM-0161) according to the manufacturer’s instructions.

### Subcellular fractionation

Cells were collected, washed with PBS, and resuspended in homogenization buffer (0.225 mol/L mannitol, 0.075 mol/L sucrose, and 20 mmol/L HEPES, pH 7.4; ~3 × 10^7^ cells/mL). Resuspended cells were processed in a Dounce homogenizer 30 times on ice. Nuclei were pelleted twice at 800 ×*g* for 5 min at 4°C. The supernatant was then centrifuged at 21,000 ×*g* for 30 min at 4°C. The supernatant from this centrifugation was used as the cytosol. For isolation of the cytoplasm, washed cells were lysed with Buffer A (10 mmol/L HEPES, pH 7.9, 10 mmol/L KCl, 1.5 mmol/L MgCl_2_, and 0.5% NP-40) on ice for 20 min and then centrifuged at 15,000 ×*g* for 10 min at 4°C. The supernatant was used as the cytoplasm. Protease inhibitors and 100 U/mL Ribolock RNase inhibitors were added during subcellular fractionation.

### Electrophoretic mobility shift assay

For EMSA assay, 4 ng biotin-labeled *Terc-53* was incubated at 65°C for 10 min and then cooled down to 4°C slowly. The isolated cytosol or purified protein was incubated with the biotin-labeled RNA in the binding buffer (125 mmol/L Tris-HCl, pH 8.0, 750 mmol/L KCl, 25 mmol/L EDTA, pH 8.0, 2.5 mmol/L DTT, 2.5% NP-40, and 100 U/mL RNase inhibitor) for 1 h at 4°C or for 30 min at 37°C. The sample was then mixed with RNA loading buffer, and resolved on the TBE-polyacrylamide gel that was per-run without samples for 30 min at 160 V in 0.5× TBE buffer. Free RNA and protein–RNA complexes were detected using Biotin detection reagents (Thermo. Cat# 89880).

### *In vitro* transcription

RNAs were synthesized using the MEGAscript SP6 Kit (Thermo, Cat# AM1320) according to the manufacturer’s instructions. Biotin RNA labeling mix (Roche, Cat# 11685597910) was used to label RNA for Northern blot or RNA pulldown. The Digoxin labeling mix (Roche, Cat# 11277073910) was used to label RNA for RNA-FISH to exclude signal interference from biotinylated proteins. RNAs were purified using TRIzol reagent. *In vitro* transcribed RNAs were treated with RNA 5’ pyrophosphohydrolase (RppH, NEB, Cat# M0356S) to remove pyrophosphate from the RNAs, and then purified by ethanol precipitation.

### RNA isolation

RNAs were purified using 400 μL TRIzol reagent and treated with 500 U RNase-free DNase I in 50 μL DNase buffer for 25 min at 37°C. DNase I was inactivated with the addition of 5 mmol/L EDTA and incubation at 70°C for 10 min. RNAs were then isolated again using TRIzol. For purification of mouse tissue RNAs, tissues were sheared into small pieces and then homogenized with a dounce homogenizer, before the TRIzol RNA purification steps.

### RT-PCR and RT-qPCR

Total RNA of 1 μg was reverse-transcribed using PrimeScript™ RT reagent Kit with gDNA Eraser (Takara, Cat# RR047B). Bio-Rad qPCR SYBR Green Master Mix was used for qPCR. 0.2 μL of the 20 μL cDNA was used for a 20 μL RT-PCR reaction. The mRNA levels were normalized by *Actb*. Oligonucleotides sequences are listed in Table S1.

### RNA pulldown

RNA pulldown was performed as previously described with minor modification (Wang et al., 2023). N2a cells in a 10 cm dish were harvested and lysed on ice in cell lysis buffer (10 mmol/L Tris, pH 7.4, 10 mmol/L KCl, 1.5 mmol/L MgCl_2_, and 1% NP-40) containing protease inhibitors and Ribolock RNase inhibitors (100 U/mL). Before the pulldown, the cytosolic lysate was incubated with streptavidin beads for 30 min to remove endogenous biotinylated proteins. Biotin-labeled (5 μg) *Terc-53* and *Terc-53* Scrambled RNA (*Scramble*) were incubated at 65°C for 10 min, cooled down slowly to room temperature, and then incubated with biotin-cleared cytosol for 2 h at 4°C, before 30 μL washed streptavidin beads was added for additional 1 h incubation. After the incubation, the beads were washed three times with wash buffer (50 mmol/L Tris-HCl, pH 7.4, 0.4 mol/L NaCl, 1 mmol/L EDTA, 1 mmol/L DTT, 0.1% NP-40, and 10% glycerol). SDS loading buffer was then added to the beads, and the protein-biotinylated RNA complexes were analyzed by immunoblotting or mass spectrometry. The peptide information islisted in Tables S2 and S3.

### RNA immunoprecipitation

N2a cells expressing Myc-tagged Hmmr or HA-tagged Trim25 were cross-linked with ultraviolet light (254 nm, 300 mJ/cm^2^). Cells were harvested and lysed in IP lysis buffer (50 mmol/L Tris-HCl, pH 7.4, 0.4 mol/L NaCl, 1 mmol/L EDTA, 1 mmol/L DTT, 1% NP-40, and 10% glycerol) containing proteinase inhibitors (1×) and RNase inhibitor (100 U/mL) for 30 min on ice. For endogenous RIP, the Hmmr commercial antibody and protein G-coupled sepharose beads were added to the cell lysate, and incubated for 2 h or overnight at 4°C. The beads were washed 3 times with IP wash buffer (50 mmol/L Tris-HCl, pH 7.4, 0.4 mol/L NaCl, 1 mmol/L EDTA, 1 mmol/L DTT, 0.1% NP-40, and 10% glycerol). RNAs co-precipitated with Hmmr, Myc-Hmmr or HA-Trim25 were purified using TRIzol and then examined by RT-PCR. The detailed information of the antibodies is listed in Table S1.

### Dot blot

Total of 1 ng *in vitro* transcribed RNA was dotted on Hybond N^+^ membrane and immunoblotted with HRP-specific streptavidin antibody.

### Co-immunoprecipitation

Cells were lysed in 300 μL IP lysis buffer (50 mmol/L Tris-HCl, pH 7.4, 10 mmol/L KCl, 1.5 mmol/L MgCl_2_, 1% NP-40) containing proteinase inhibitors (1×) and RNase inhibitors (100 U/mL) for 30 min on ice. The lysate was centrifuged at 13,000 ×*g* for 30 min. 2% of the supernatant was kept as the input. The remaining supernatant was used for immunoprecipitation with Myc-beads or Protein G/A-coupled Sepharose beads pre-incubated with the intended antibody. After incubation at 4°C overnight, the beads were washed three times with IP lysis buffer. 2× SDS loading buffer was added to the beads and the samples were analyzed by immunoblotting.

### SDS-PAGE and immunoblotting analysis

Total proteins or the cytosolic proteins were extracted from cells and tissues using radio-immunoprecipitation assay (RIPA) buffer (150 mmol/L NaCl, 50 mmol/L Tris-HCl, pH 8.0, 1% Nonidet P-40, 0.5% sodium deoxycholate, 0.1% SDS), supplemented with protease inhibitors (1:100, MCE). The protein concentration was measured using BCA Protein Assay Kit (Beyotime). Equal amounts of proteins were separated by electrophoresis on 8%–12% polyacrylamide gels, transblotted onto a polyvinylidene difluoride membrane (PVDF). and incubated with antibodies against Hmmr (1:1000, abcam, Cat# ab124729), Trim25 (1:1000, Proteintech, Cat#12573-AP), GluA1 (1:1000, CST, Cat# 13185), Bdnf (1:500, Abclonal, Cat# AP4873), p65(1:1000, Abclonal, Cat# A22676), P-p65 (1:500, Abclonal, Cat# AP0475), HA (1:10,000, Abclonal, Cat# AE008), Flag (1:5,000, abcam, Cat# ab205606), Myc (1:10,000, Abclonal, Cat# AE010), β-Tubulin (1:5,000, CST, Cat# 2128) or β-actin (1:5,000, CST, Cat#3700) at room temperature for 1 h or at 4°C overnight. After three wash steps, the membrane was incubated with HRP-conjugated anti-rabbit IgG (1:10,000, CST, Cat# 7074S) or anti-mouse IgG (1:10,000, CST, Cat# 7076S) for 1 h at room temperature. In some experiments, the previous primary antibody and secondary antibody were stripped from the PVDF membrane with stripping buffer (Beyotime, Cat# P0025) for 10 min at room temperature. After being re-blocked, the membrane was re-incubated with another primary antibody at 4°C overnight. The following steps were the same as described above. Immunoreactive bands were visualized using Bio-Rad Image Lab. The antibody information was listed in Table S1.

### Northern blot

Northern blot was performed as previously described, with North2South Hybridization and Detection Kit (Thermo, Cat# 17097). For *Terc-53*, *Terc* and *5S* rRNA, 20 µg cytosolic RNA, 10 µg nuclear RNA, and 0.5 µg cytosolic RNA respectively were loaded onto 6%–12% polyacrylamide gels containing 8 mol/L Urea, run at 160 V for 60 min, and transferred to Amersham Hybond N+ membrane (GE) at 400 mA for 90 min. The membrane was hybridized with biotin-labeled RNA probes at 65°C overnight. Biotin-labeled *Terc-53* antisense RNA probe was used to detect the expression of *Terc-53*. For *5S* rRNA, biotin-labeled full-length *5S* antisense RNA probe was used. For *Terc*, biotin-labeled full-length antisense RNA probe was used. The probe information is listed in Table S1.

### RNA fluorescence *in situ* hybridization

RNA FISH and co-immunofluorescent staining of cells or tissues was performed as previously described (Zhao et al., 2020) with minor modification. In brief, cells or brain slices were fixed in 4% formaldehyde and 5% acetic acid followed by 3 washes with PBS. The fixed cells were further treated with pepsin (1% in 10 mmol/L HCl) and dehydrated in 70%, 90%, and 100% ethanol. The air-dried cells were then subjected to incubation with 40 nmol/L *Terc-53* FISH probe in hybridization buffer (100 mg/mL dextran sulfate, 10% formamide in 2× SSC) at 80°C for 2 min. The slices or the cells were rehydrated in 50% formamide in 2× SSC for 5 min and prehybridized in 2× SSC for 1 h at 52°C. The cells were then hybridized with digoxin-labeled oligonucleotide probes against *Terc-53* as described above. After hybridization, endogenous peroxidase activity was blocked with 3% H_2_O_2_. The samples were washed with 5× SSC, 2× SSC in 0.1% PBST for 5 min, and then incubated with anti-digoxigenin-FITC antibody (1:200, Roche, Cat# 11207741910), anti-mouse Hmmr antibody (1:200, Santa Cruz, Cat# sc-515221), or/and anti-rabbit Trim25 (1:200, Proteintech, Cat#12573-AP) at room temperature for 1 h. The samples were then washed with PBST and incubated with Alexa Fluor 594 donkey anti-mouse IgG or/and Alexa Fluor 647 ab150083 donkey anti-rabbit IgG at room temperature for 1 h, before being stained with DAPI. Cell signals were quantitated per field of view, and at least 10 fields per section were evaluated using a confocal microscope (Zeiss LSM 980). Zeiss 3.1 (blue edition) software was used to evaluate the colocalization of *Terc-53* signals and Hmmr. Colocalization percentage was calculated by dividing the number of Alexa Fluor 594 and FITC colocalizing spots by the number of FITC positive spots. Percentage of *Terc-53*+, NeuN+, IBA1+, and GFAP+ colocalization signals was evaluated in a similar manner. At least 10 view-fields per sample were evaluated and quantitated.

### Protein purification

His-Hmmr protein was expressed in bacteria BL21 (DE3). Protein expression was induced with IPTG (1 mmol/L) in 100 mL of bacterial culture at 18°C for 12 h. The bacteria were collected, and sonicated in 4 mL buffer A (20 mmol/L HEPES pH 7.4, 300 mmol/L NaCl, 20 mmol/L imidazole, 5% glycerin, 1 mmol/L DTT) with protease inhibitors (MCE). Insoluble materials were removed by centrifugation and the extract was transferred to a new tube. 50 µL Ni^2+^ NTA resin (QIAGEN, Cat# 30230) was added to 1 mL of the extract for 2 h incubation at 4°C. After the incubation, the resin was washed three times with protease inhibitor-containing buffer A. Proteins on the beads were eluted with protease inhibitor-containing Buffer B (20 mmol/L HEPES pH 7.4, 150 mmol/L NaCl, 400 mmol/L imidazole, 5% glycerin, 1 mmol/L DTT, 0.05% DDM). The concentration of the eluates was measured using BCA Protein Assay Kit, and the proteins within were examined by immunoblotting.

### Examination of ubiquitination types and levels

Cells were treated with 20 μmol/L MG132 (MCE, Cat# HY-13259) for 4 h, 50 μg/mL cycloheximide (Amresco, Cat# 0528C138) for 3 h or 25 μmol/L chloroquine (Macklin, Cat# C798394) for 12 h, and then collected and lysed in IP lysis buffer (50 mmol/L Tris-HCl, pH 7.4, 10 mmol/L KCl, 1.5 mmol/L MgCl_2_, 1% NP-40) supplemented with 1 mmol/L PMSF. Cell lysates were denatured by boiling at 95°C for 5 min in the presence of 1% SDS, and then cooled down on ice for 3 min, followed by centrifugation at 1000 ×*g* at room temperature for 5 min. The supernatant was diluted 10 times with lysis buffer and subject to immunoprecipitation with the indicated antibodies and analyzed by immunoblotting.

### GEO data analysis

The published datasets GSE118553 were downloaded from the Gene Expression Omnibus (GEO) for *in silico* analysis of *HMMR* gene expression in 22 control, and 32 AD subjects. Data generated with tissues known to be affected by AD neuropathology (entorhinal cortex) and tissues partially spared by the disease (cerebellum) were analyzed.

### RNA sequencing (RNA-seq) and data analysis

The cDNA libraries were constructed from 1 μg RNA sample using an ABclonal Whole RNA-seq Lib Prep kit for illumina (ABclonal, Cat# RK20351) following the guidelines of the manufacturer. The concentrations of cDNA libraries were measured using Qubit dsDNA HS Assay Kit. Sequencing was performed on an Illumina Novaseq 6000 instrument (150 bp, paired-end) at N6ovogene Co., Ltd. (Beijing, China). Basecalls of millisect RNA-seq data were performed using CASAVA (v.1.8). Purity-filtered reads were trimmed with Cutadapt (v.1.18) (DOI: doi.org/10.14806/ej.17.1.200). Reads were aligned against the mm10 mouse reference genome using STAR (v.2.5.2b) (Dobin et al., 2013). The number of read counts per gene locus was summarized with GenomicAlignments (v.1.22.1) (Lawrence et al., 2013). Differential expression analysis was performed with DESeq2 (v.1.24.0) (Love et al., 2014) based on the negative binomial distribution, using default parameters. Significance tests for multiple comparisons were performed by Benjamini & Hochberg correction. Pheatmap (v.1.0.12) was used for the visualization of heatmaps of genes selected according to their functions. Colors represent the fold change values (log2 scale) calculated from the regularized, log2-transformed gene FPKM value obtained from DESeq2 (v.1.24.0). RNA-seq data including in Tables S4 and S5.

### Telomerase activity and telomere length measurement

Telomere repeat amplification protocol (TRAP) and TRAPeze® Telomerase Detection Kit (Millipore, Cat. No. S7700) were used for testing telomerase activity. Mouse tissues were collected and used for the analysis of telomerase activity. The PCR products were resolved on agarose gels (3%) and visualized by staining with gel red Safe. Telomere length was measured from mouse brain cortex genomic DNA using a real-time qPCR method and calculated using telomere PCR (T)/single copy gene PCR (S) as previously described (Cawthon, 2002; Wang et al., 2022). Real-time qPCR primers are shown in Table S1. The PCR reaction conditions for telomere were 95°C for 10 min and 30 cycles of 95°C for 15 s, and 56°C for 1 min. The reaction conditions for *36B4* housekeeping gene were 95°C for 10 min and 35 cycles of 95°C for 15 s, 52°C for 20 s, and 72°C for 30 s. Each reaction of the assay consisted of 5 μL SYBR qPCR Master Mix, forward and reverse primers (concentrations as described before), 50 ng genomic DNA, and double-distilled H_2_O to yield a 10 μL reaction.

### Animals and generation of G2 *Terc*^−/−^, *Terc*^−/−^-*Terc-53,* and *Terc*^−/−^-*Terc-53 AS* mice

Sanger sequencing of PCR products was used to characterize the *Terc-53* and *Terc-53* antisense (*Terc-53 AS*) clones. Purified DNA was microinjected into pronuclei of C57BL/6 fertilized oocytes and transplanted into pseudopregnant foster mothers. The transgenic offspring were screened by PCR. Transgenic mice, Tg-*Terc-53* and Tg-*Terc-53 AS* mice were maintained in a C57BL/6 background.

C57BL/6 *Terc*^+/−^ heterozygous female and male mice were intercrossed to obtain first-generation (G1) *Terc*^−/−^ litters. G1 *Terc*^−/−^ mice were then intercrossed to obtain second-generation (G2) *Terc*^−/−^ offspring.

*Terc* ^−/−^ *-Terc-53* and *Terc*^−/−^*-Terc-53 AS* mice were obtained by crossing *Terc*^−/−^ mice with *Terc*^+/−^*-Terc-53* and *Terc*^+/−^*-Terc-53 AS* mice.

The 5× FAD mice expressing five familial AD gene mutations (stock no. 34840-JAX) were obtained from the Jackson Laboratory (Ellsworth, ME, USA).

Genotype was confirmed by using PCR. The primers used are listed in Table S1.

For LPS treatment, age-matched male *Terc*^−/−^ mice were intraperitoneally injected with LPS (Amresco, Cat# AC11974) dissolved in sterile 0.9% saline at 2 mg/kg. This dosage was used to stimulate microglial activation without other obvious maladies. LPS injection was administered between 09:00 and 09:30 a.m. daily for 4 days.

Age-matched male mice were exclusively used for behavioral tests. Electrophysiology examination was used to exclude interfering effects derived from estrogen. Body weight was measured weekly or monthly.

### Rotarod test

An accelerated Rota (Ugo Basile 47650) was used for the test. Mice were acclimated to the treadmill 2 days prior to the experiments by running for 5 min/day at 5 m/min and 10 m/min followed by 15 m/min for 1 min until exhaustion. For test experiments, speed was increased 5 m/min every 5 min, and the revolutions per minute (rpm) of mice to fall was recorded. Four consecutive trials were repeated, and averaged latency to fall was analyzed.

### Open field

The open field test was used to assess locomotor activity. Prior to the behavioral test, mice were allowed to adapt to the test room for at least 2 days (at least 4 h per day). Each mouse was placed in a square open field box (50 × 50 cm) that was divided into peripheral and central zones with walls 35 cm high. The test began by gently placing the mouse into a corner of the box and the total distance travelled, time spent in the inner zones were observed for 5 min duration by a video camera. Noldus EthoVision XT software was used to record and analyses locomotor activity of mice.

### Morris water maze

The water maze used in this study comprised a circular tank 120 cm in diameter with a platform filled with tap water at a temperature of 22 ± 2°C. Different shapes were posted along the walls of the tank, which served as spatial reference cues. A camera was mounted above the maze to record the swimming traces in the water maze. During the acquisition trials, the platform was submerged 1–2 cm below the water surface. Mice were placed into the maze at one of four points (N, S, E, and W) facing the wall of the tank. Mice were allowed to search for the platform for 60 s. If a mouse failed to find the platform, it was guided to the platform and maintained on the platform for 10 s. Four trials a day were conducted with an intermission of 1 h minimum between the trials. Escape latency indicative of spatial memory acquisition was recorded for each trial. On day 6, the platform was removed and a probe test was conducted. The percentage time spent in each of the four quadrants and the number of target (platform) area crossings, mean speed, and total distance were recorded. Noldus EthoVision XT software was used to record and analyses the locomotor activity of mice.

### Y maze

For Y-maze spontaneous alternation analysis, mice were housed and allowed to acclimate in the testing room for 72 h. A Y-shaped maze comprises three identical arms. A mouse, placed at the center of the maze, was allowed to freely explore the maze for 5 min. During the test period, entries of limbs that pass through all arms were recorded with a video camera (Noldus EthoVision XT) for 10 min. An arm entry occurred when all four limbs were within the arm. Total arm entries and spontaneous alternation (%) in three different arms were recorded and analyzed. Alternation (%) was defined as consecutive entries in three different arms, divided by the number of possible alternations (total arm entries minus 2).

### Generation and delivery of adeno-associated virus (AAV)

Generation and delivery of recombinant adeno-associated virus (AAV) was performed as previously described with minor modification (Shi et al., 2021). This system contains a transgene plasmid with a promoter and target cDNA placed between the two 145-base ITRs (from type 2 AAV), a transfer plasmid with sequences coding for REP (from type 2 AAV) and CAP (from type 9 AAV), and a helper plasmid with E4, E2a, and VA (from adenovirus). The promoter of Hsyn was used for neuron-specific expression of genes. The cells and the medium containing packaged viruses were collected 60–72 h after transfection. 5× polyethylene glycol (PEG; 40% PEG-8000, 2.5 mol/L NaCl) was added to the medium to a final concentration of 8% PEG-8000 and 0.5 mol/L NaCl, and the samples were incubated overnight at 4°C. After the incubation, the mixture was centrifuged at 2,818 ×*g* for 5 min at 4°C. The pellet containing the virus was saved. The cells were lysed in lysis buffer (150 mmol/L NaCl, 20 mmol/L Tris pH 8.0) through three rounds of freezing in liquid nitrogen, followed by thawing in a 37°C incubator. The mixture was then centrifuged at 3,220 ×*g* for 10 min at 4°C, and the supernatant containing the virus was saved. The supernatant from the cell lysate and the pellet from the medium were then mixed and layered on a 17%, 25%, 40%, and 60% iodixanol (Sigma-Aldrich) gradient for a 75 min ultracentrifugation at 60,000 rpm (Beckman NVT65 or 70.1Ti) and 16°C. The viruses were extracted from the 40% iodixanol gradient, washed three times with PBS in the ultrafiltration, and pelleted at 3,500 rpm for 30 min at 4°C. The copy number of AAV was determined by quantitative PCR (Bio-Rad, CFX96). The primers used are listed in Table S1.

For virus delivery, G2 *Terc*^−/−^ or *Terc*^−/−^*-Terc-53* mice were fixed on the brain stereotactic apparatus (RWD Life Science) horizontally and anesthetized with isoflurane gas. A longitudinal incision was made to expose the skull surface. The anterior fontanelle was set as the origin of the coordinates and a hole was drilled with a skull drill (RWD Life Science), according to the ventral hippocampal coordinates. A micro-syringe (RWD Life Science Co., Ltd., Shenzhen, China) was positioned above the drilled hole according to the coordinates and was used to take in the AAV virus (titer = 5 × 10^12^ IU/mL), then the injection needle was slowly lowered through the hole into the target brain area. A small volume of virus was injected bilaterally into the hippocampus (AP, −1.9 mm from bregma; DV, −1.5 mm from skull surface; ML, ± 1.15 mm from bregma) using a pulled glass capillary with a pressure microinjector at a slow rate of 50 nL/min. Five minutes later, a syringe pump controller was used (RWD Life Science) to perform a bilateral injection of 1 µL per side at a speed of 2 nL/s. After viral injection, the needle was left for an additional 5 min and then slowly removed. The scalp was sutured and the mice were then returned to the cage after awakening.

### Electrophysiology

After a mouse were anesthetized with isoflurane, the brain was dissected rapidly and placed in ice-cold artificial hypertonic glucose solution (10 mmol/L D-glucose, 120 mmol/L sucrose, 10 mmol/L MgSO_4_, 2.5 mmol/L KCl, 1.25 mmol/L NaH_2_PO_4_, 26 mmol/L NaHCO_3_, 0.5 mmol/L CaCl_2_, and 64 mmol/L NaCl). The brain was cut coronally (400 mm thick) with Leica VT1200S vibratome in artificial hypertonic glucose solution bubbled with carbogen (95% O_2_ + 5% CO_2_). The slices were incubated in ACSF (120 mmol/L NaCl, 1.3 mmol/L MgSO_4_, 3.5 mmol/L KCl, 10 mmol/L D-glucose, 2.5 mmol/L CaCl_2_, 1.25 mmol/L NaH_2_PO_4_, and 26 mmol/L NaHCO_3_), bubbled with carbogen at 32°C for 1 h and at room temperature for at least another 1 h.

Schaffer collateral inputs to the CA1 region of hippocampus were selected for stimulation using a bipolar stimulating electrode (FHC, Inc.) with glass micropipettes (13 MU) filled with ACSF. The signals were recorded by Multi-Clamp 700B amplifier (Molecular Devices) and digitized using Digidata 1550 B. The slice was transferred to a recording chamber perfused with ACSF (2 mL/min). A bipolar tungsten electrode was used to stimulate Schaffer collateral inputs to the CA1 region, and field excitatory postsynaptic potentials (fEPSPs) were recorded in the CA1 dendritic layer. fEPSP responses were evoked every 20 s and recorded at a stimulus intensity of 10–700 μA. Based on the stimulus response curve, the stimulation intensity at 30% of the maximum response was used for baseline measurements. After a stable fEPSP response was recorded for at least 20 min, high-frequency stimulation (2 trains, 100 Hz, 30 s intervals) was applied to induce LTP, and fEPSPs were recorded for another 60 min. The data were filtered at 2 kHz, sampled at 10 kHz, collected with a Multiclamp 700B amplifier and analyzed with pClamp 10.6 software (Molecular Devices). The changes in LTP were calculated as the fEPSP slope (after LTP induction) minus the baseline fEPSP slope.

### Enzyme linked immunosorbent assay (ELISA)

Il-1β and Ccl7 expression levels in brain were measured using mouse Il-1β, Ccl7 ELISA Kits (Neobioscience, mouse Il-1β ELISA kit, EMC001b and Solarbio mouse-Ccl7 ELISA Kit, SEKM-0161) according to the manufacturer’s instructions.

### Statistical analysis

All data presented are presented as arithmetic mean ± SD if not otherwise specified in figure legend as mean ± SEM. All statistical analyses were performed using GraphPad Prism. For normally distributed data, we used unpaired Student’s *t* tests to evaluate statistical significance of differences between the two groups. Statistically significant differences between groups were determined using one-way ANOVA.

All electrophysiological results were analyzed using Sigma Stat 4 statistical software. Statistical significance was evaluated by oneway ANOVA with Holm-Sidak pairwise tests. Values of *P* < 0.05 were considered to be statistically significant. SnapGene software was used to analyze Sanger sequencing data.

## Supplementary information

The online version contains supplementary material available at https://doi.org/10.1093/procel/pwae023.

pwae023_suppl_Supplementary_Material

pwae023_suppl_Supplementary_Table_S1

pwae023_suppl_Supplementary_Table_S2

pwae023_suppl_Supplementary_Table_S3

pwae023_suppl_Supplementary_Table_S4

pwae023_suppl_Supplementary_Table_S5

## Data Availability

All data associated with this study are presented in the paper or the Supplementary Materials. The software used in the current study has been cited in “Methods” section. Please address all requests for reagents and materials to G.W. (wangengfuan@xmu.edu.cn). All data needed to evaluate the conclusions in the paper are present in the paper and/or Supplementary Materials.

## Code availability

This paper does not report original code.
