# Supplementary material for: Noncoding RNA Terc-53 and hyaluronan receptor Hmmr regulate aging in mice
Source: Protein Cell. 2024 May 9;16(1):28–48. doi: 10.1093/procel/pwae023 (PMC11700592; doi:10.1093/procel/pwae023)
Supplement: pwae023_suppl_Supplementary_Material [file pwae023_suppl_supplementary_material.pdf]

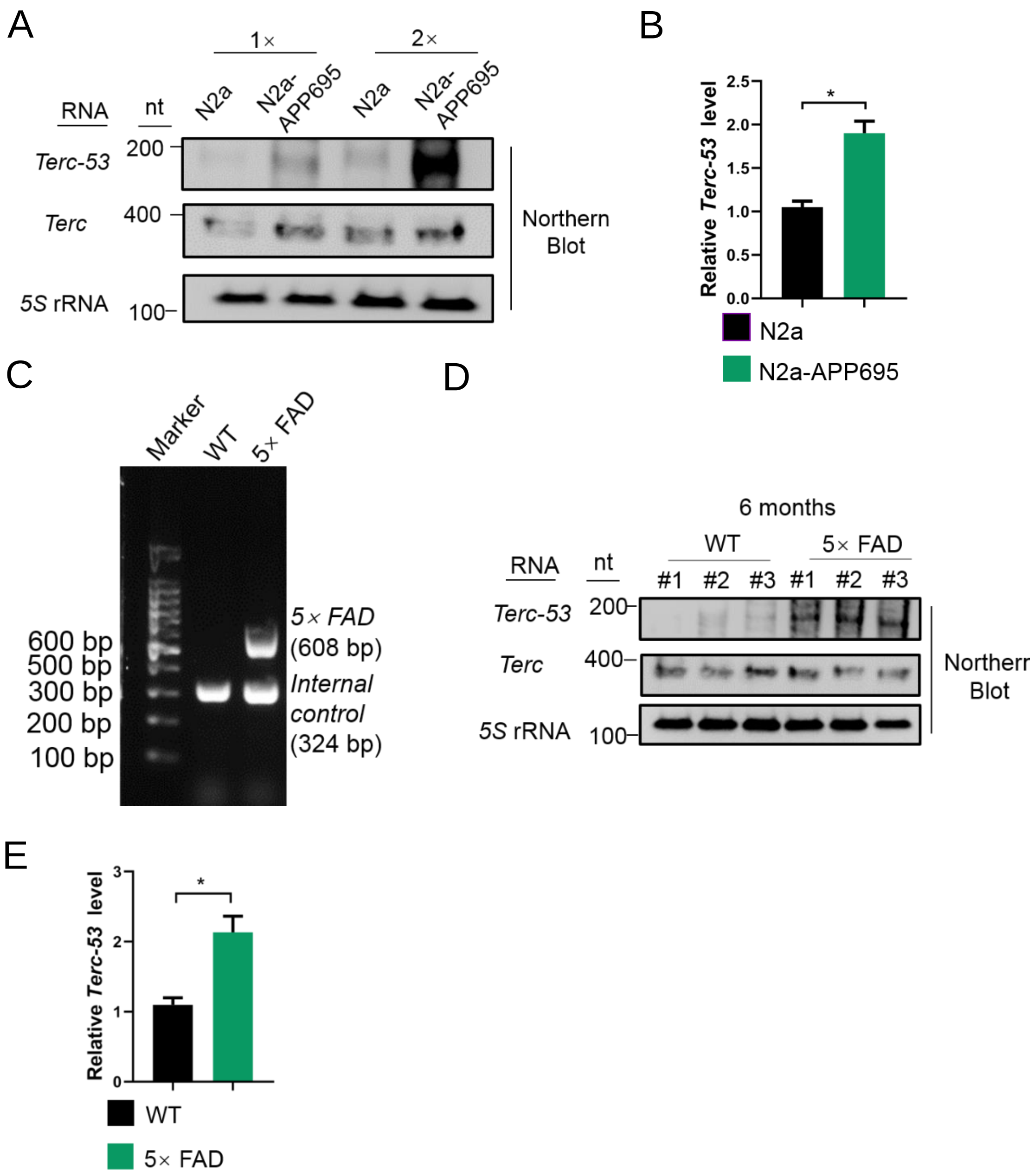

Figure S1. Association of *Terc-53* with Alzheimer's disease.

(A) Northern blotting examination of *Terc-53*, and full-length *Terc* in N2a and N2a-APP695 cells. *5S* rRNA was used as a loading control.

(B) Quantification of the relative *Terc-53* levels in Panel A (n = 3).

(C) Genotyping of day 21 WT and 5× FAD mice.

(D) Northern blotting examination of *Terc-53* and full-length *Terc* in the hippocampus of 5× FAD male mice and wild-type (WT) male mice at 6 months of age. *5S* rRNA was used as a loading control.

(E) Quantification of the relative *Terc-53* levels in Panel D (n = 3).

Data are presented as Mean ± SD. ns, no significance, *$p < 0.05$, **$p < 0.01$.

Figure S2

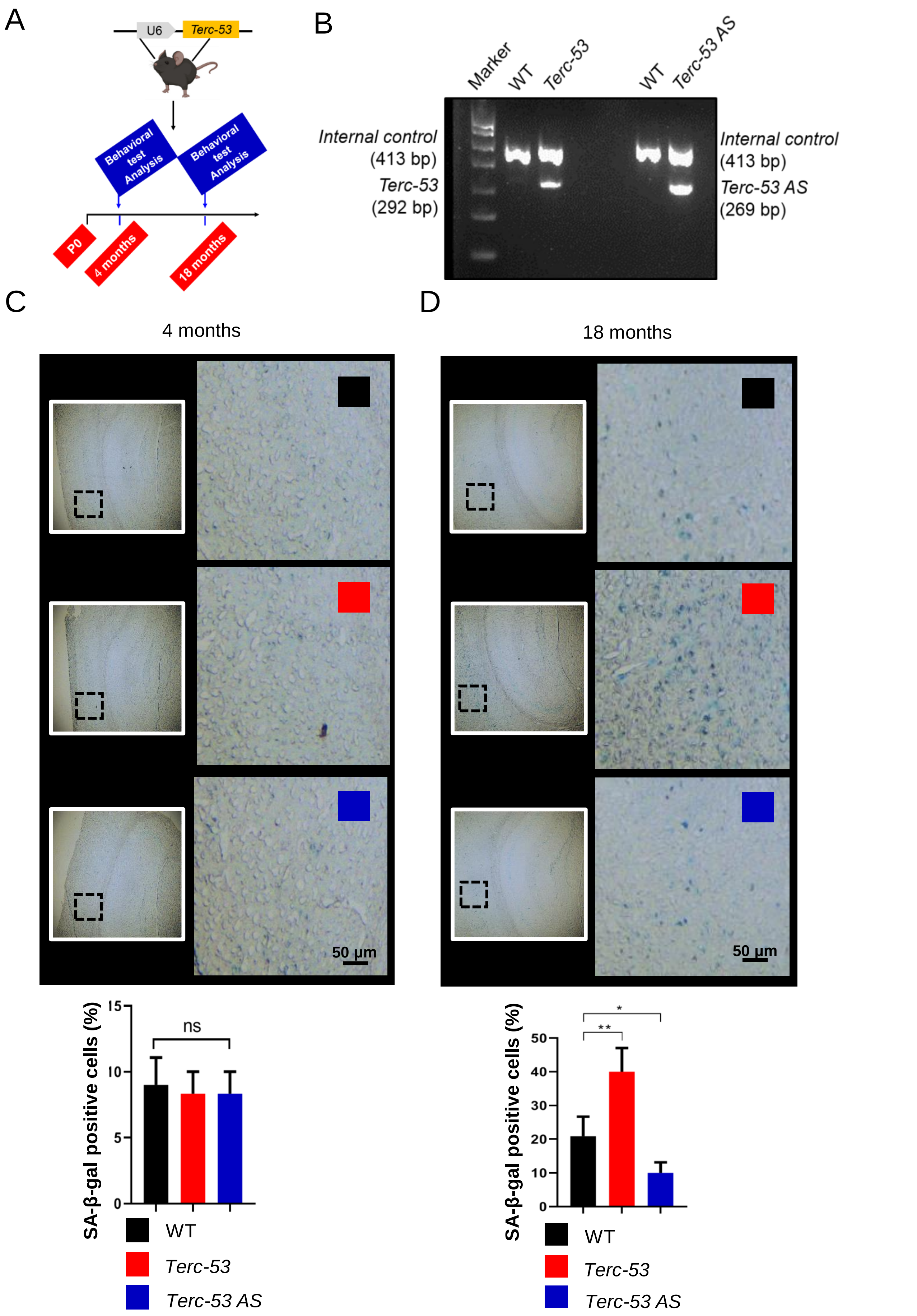
A
U6
Terc-53
Behavioral test Analysis
Behavioral test Analysis
P0
4 months
18 months
B
Marker
WT
Terc-53
WT
Terc-53 AS
Internal control (413 bp)
Terc-53 (292 bp)
Internal control (413 bp)
Terc-53 AS (269 bp)
C
4 months
50 μm
D
18 months
50 μm
SA-β-gal positive cells (%)
ns
WT
Terc-53
Terc-53 AS
SA-β-gal positive cells (%)
*
**
WT
Terc-53
Terc-53 AS

E

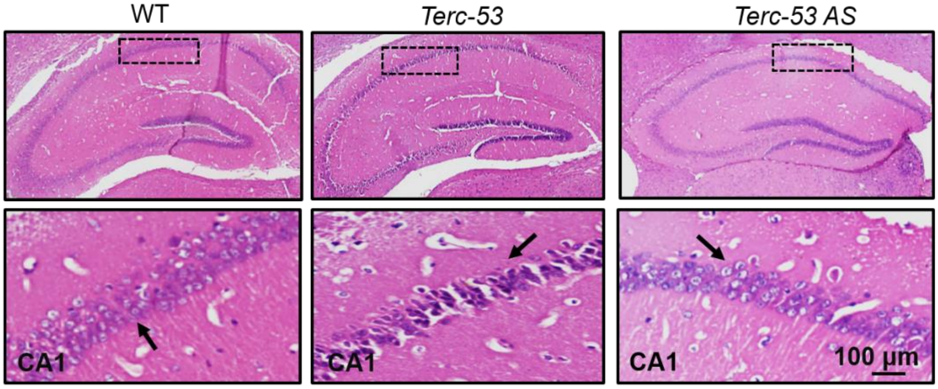

Figure S2. ***Terc-53*** **overexpression causes abnormality in the 18-month-old mouse hippocampus.**

(A) Schematic of the strategy for generating the transgenic *Terc-53*-overexpressing mice (*Terc-53*) and transgenic *Terc-53* antisense-expressing mice (*Terc-53 AS*) and the time point experimental flowchart.

(B) Genotyping of 21-day-old WT, *Terc-53* and *Terc-53 AS* mice using PCR. The higher band is an internal PCR control.

(C) Representative SA-β-gal staining images of the brain sections of WT, *Terc-53* and *Terc-53 AS* male mice at 4 months of age (top panels), and quantification of the SA-β-gal positive cells (bottom panel).

(D) Representative SA-β-gal staining images of the brain sections of WT, *Terc-53* and *Terc-53 AS* male mice at 18 months of age (top panels), and quantification of the SA-β-gal positive cells (bottom panel).

(E) Representative hematoxylin and eosin (H&E) staining images of the brain sections of WT, *Terc-53* and *Terc-53 AS* mice at 18 months of age. The nucleus shows in purple while the cytoplasm shows in red. Black arrows indicate the neurons.

Data are presented as Mean ± SD. ns, no significance, $^{*}p < 0.05$, $^{**}p < 0.01$.

Figure S3

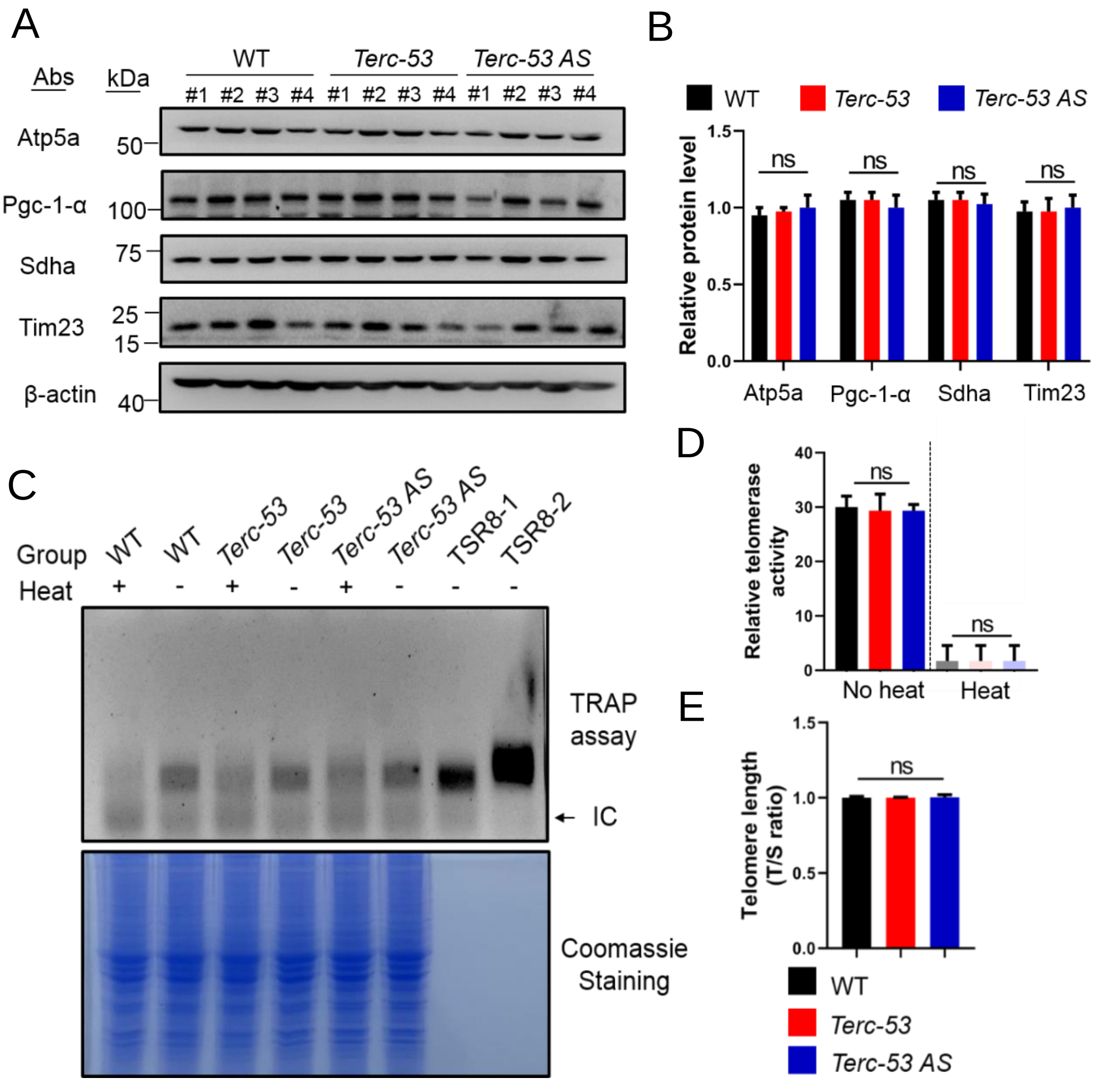

A
Abs
kDa
WT
Terc-53
Terc-53 AS
#1 #2 #3 #4 #1 #2 #3 #4 #1 #2 #3 #4
Atp5a
50
Pgc-1-α
100
Sdha
75
Tim23
25
15
β-actin
40
B
WT
Terc-53
Terc-53 AS
Relative protein level
1.5
1.0
0.5
0.0
ns
Atp5a
Pgc-1-α
Sdha
Tim23
C
Group
Heat
WT
WT
Terc-53
Terc-53
Terc-53 AS
Terc-53 AS
TSR8-1
TSR8-2
+ - + - + - - -
TRAP assay
IC
Coomassie Staining
D
Relative telomerase activity
40
30
20
10
0
ns
No heat
Heat
E
Telomere length (T/S ratio)
1.5
1.0
0.5
0.0
ns
WT
Terc-53
Terc-53 AS

Figure S3. *Terc-53* overexpression does not affect mitochondrial biosynthesis and functions or telomerase activity.

(A) Immunoblots of the proteins that are involved in mitochondrial biosynthesis and functions in the hippocampus of the three groups of mice at 18 months of age. β-actin was used as a loading control.

(B) Quantification of the relative protein levels in Panel A.

(C) Measurement of telomerase activity in the hippocampus of the three groups of mice at 18 months of age by telomerase repeat amplification protocol (TRAP) assay. The bottom panel is a Coomassie staining gel of the hippocampus lysates as the loading control.

(D) Quantification of the relative telomerase activity in Panel C (n = 3).

(E) Measurement of the telomere length in the cortex of the three groups of mice at 18 months of age, using a real-time qPCR method and calculating telomere PCR (T) /single copy gene PCR (S) ratio.

Data are presented as Mean ± SEM. ns, no significance.

Figure S4

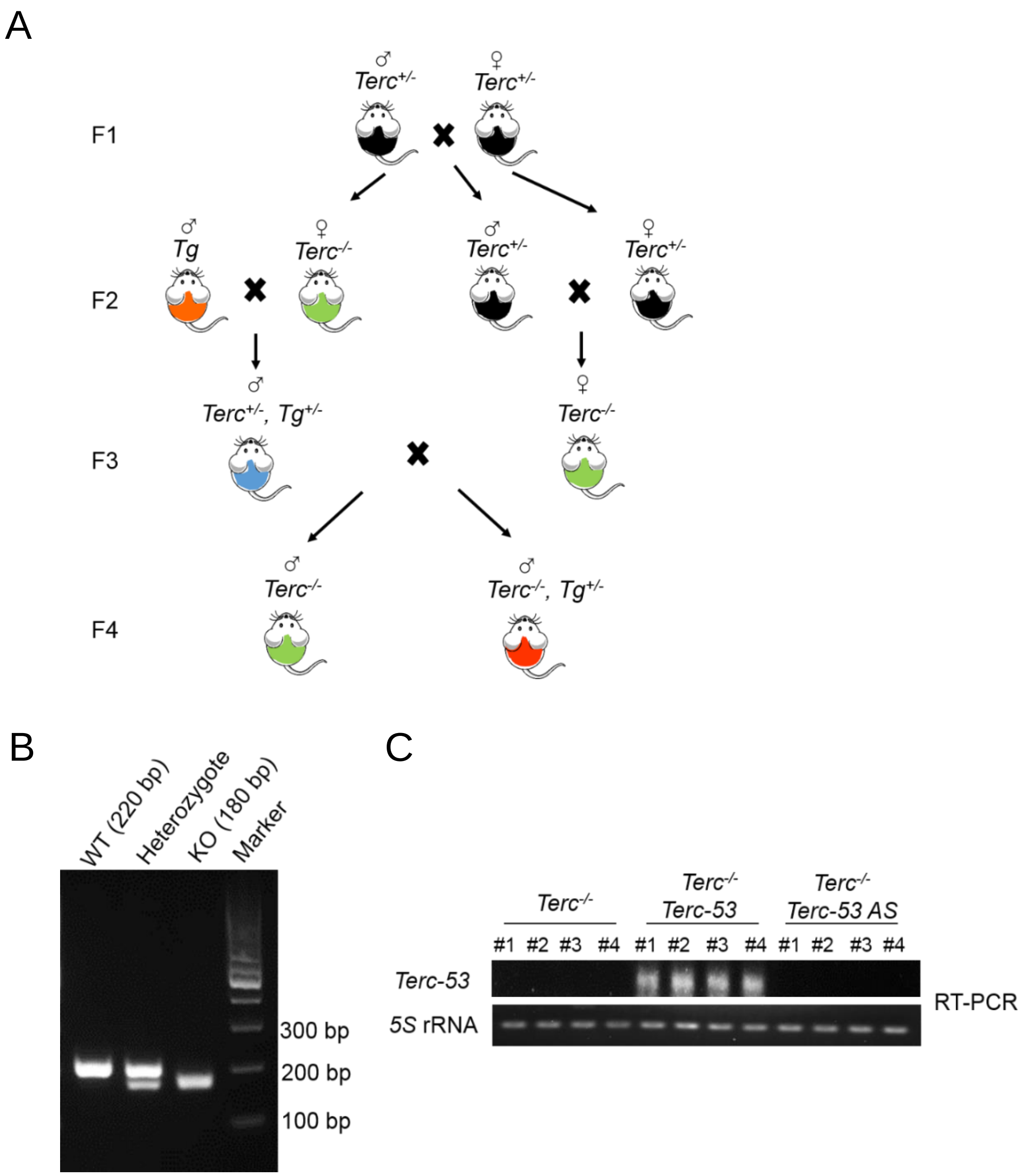

Figure S4. Generation of *Terc-53* transgenic mice on *Terc*$^{-/-}$ background.

(A) Schematic of the strategy for generating the *Terc* knockout (*Terc*$^{-/-}$), *Terc* knockout plus transgenic *Terc-53*-overexpressing mice (*Terc*$^{-/-}$-*Terc-53*), and *Terc* knockout plus transgenic *Terc-53* antisense-expressing mice (*Terc*$^{-/-}$-*Terc-53 AS*).

(B) Genotyping of 21-day-old WT, *Terc*$^{+/-}$ and *Terc*$^{-/-}$ mice.

(C) *Terc-53* levels in the cortex of *Terc*$^{-/-}$, *Terc*$^{-/-}$-*Terc-53 and Terc*$^{-/-}$-*Terc-53 AS* mice at 18 months of age were examined using RT-PCR. *5S* rRNA was used as a loading control.

Figure S5

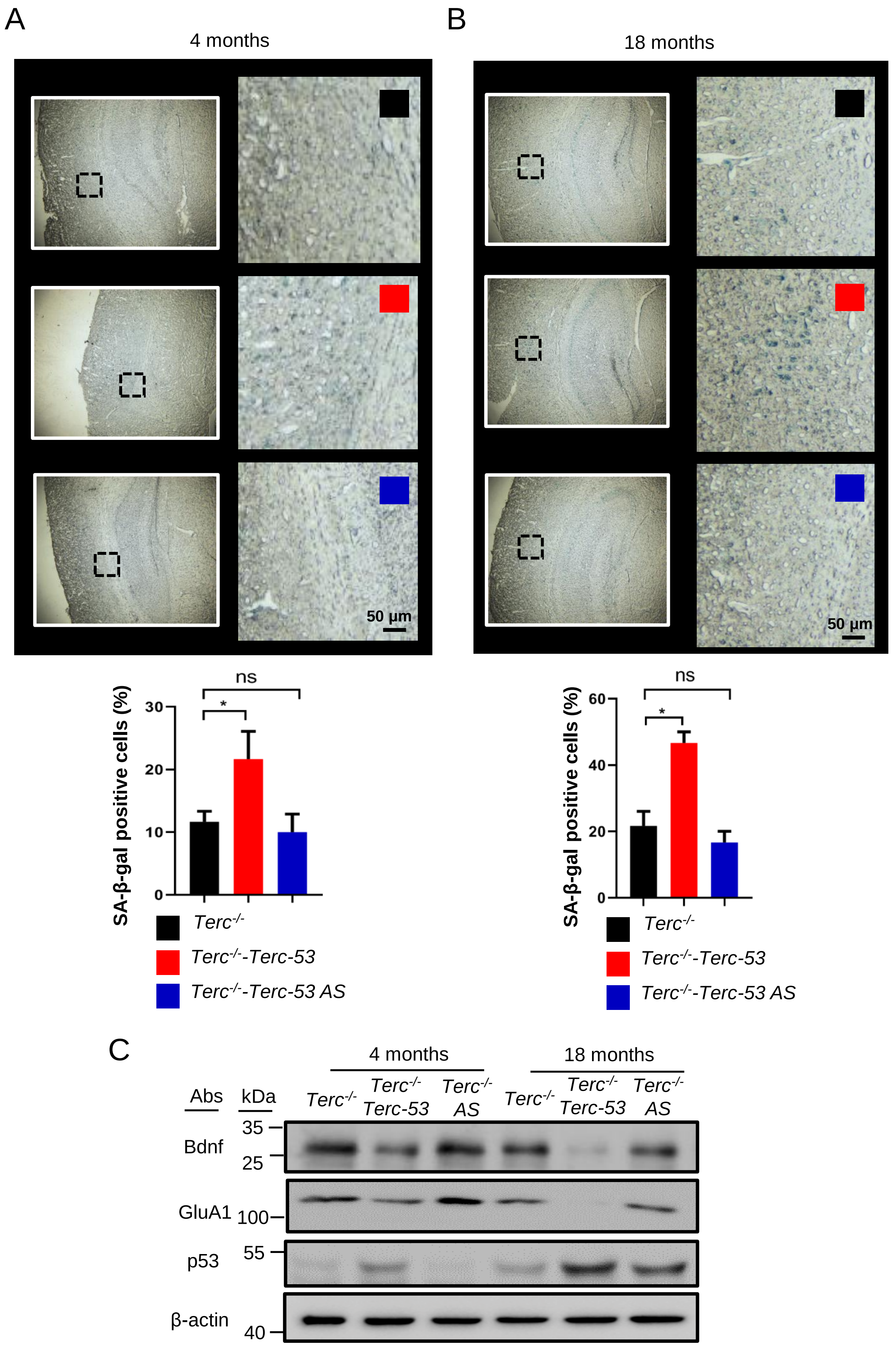

A
4 months
50 µm
B
18 months
50 µm
ns
*
SA-β-gal positive cells (%)
30
20
10
0
60
40
Terc-/-
Terc-/--Terc-53
Terc-/--Terc-53 AS
C
4 months
18 months
Abs
kDa
Terc-/-
Terc-/- Terc-53
Terc-/- AS
Bdnf
35
25
GluA1
100
p53
55
β-actin
40

D

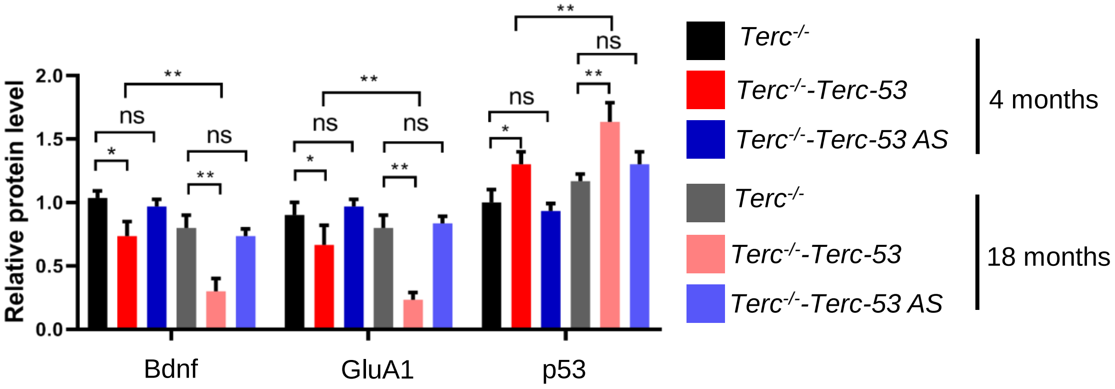

Figure S5. The effects of *Terc-53* overexpression on ageing markers in the hippocampus of *Terc*$^{-/-}$ mice.

(A) Representative SA-β-gal staining images of the brain sections of *Terc*$^{-/-}$, *Terc*$^{-/-}$*-Terc-53 and Terc*$^{-/-}$*-Terc-53 AS* male mice at 4 months of age (top panels), and quantification of the SA-β-gal positive cells (bottom panel).

(B) Representative SA-β-gal staining images of the brain sections of *Terc*$^{-/-}$, *Terc*$^{-/-}$*-Terc-53 and Terc*$^{-/-}$*-Terc-53 AS* male mice at 18 months of age (top panels), and quantification of the SA-β-gal positive cells (bottom panel).

(C) Immunoblots of Bdnf (Brain-derived neurotrophic factor) and GluA1 (an AMPA receptor subunit) in the hippocampus of the three groups of mice at 4 and 18 months of age. β-actin was used as a loading control.

(D) Quantification of the relative protein levels in Panel C (n = 4).

Data are presented as Mean ± SD. ns, no significance, *p < 0.05, **p < 0.01.

Figure S6

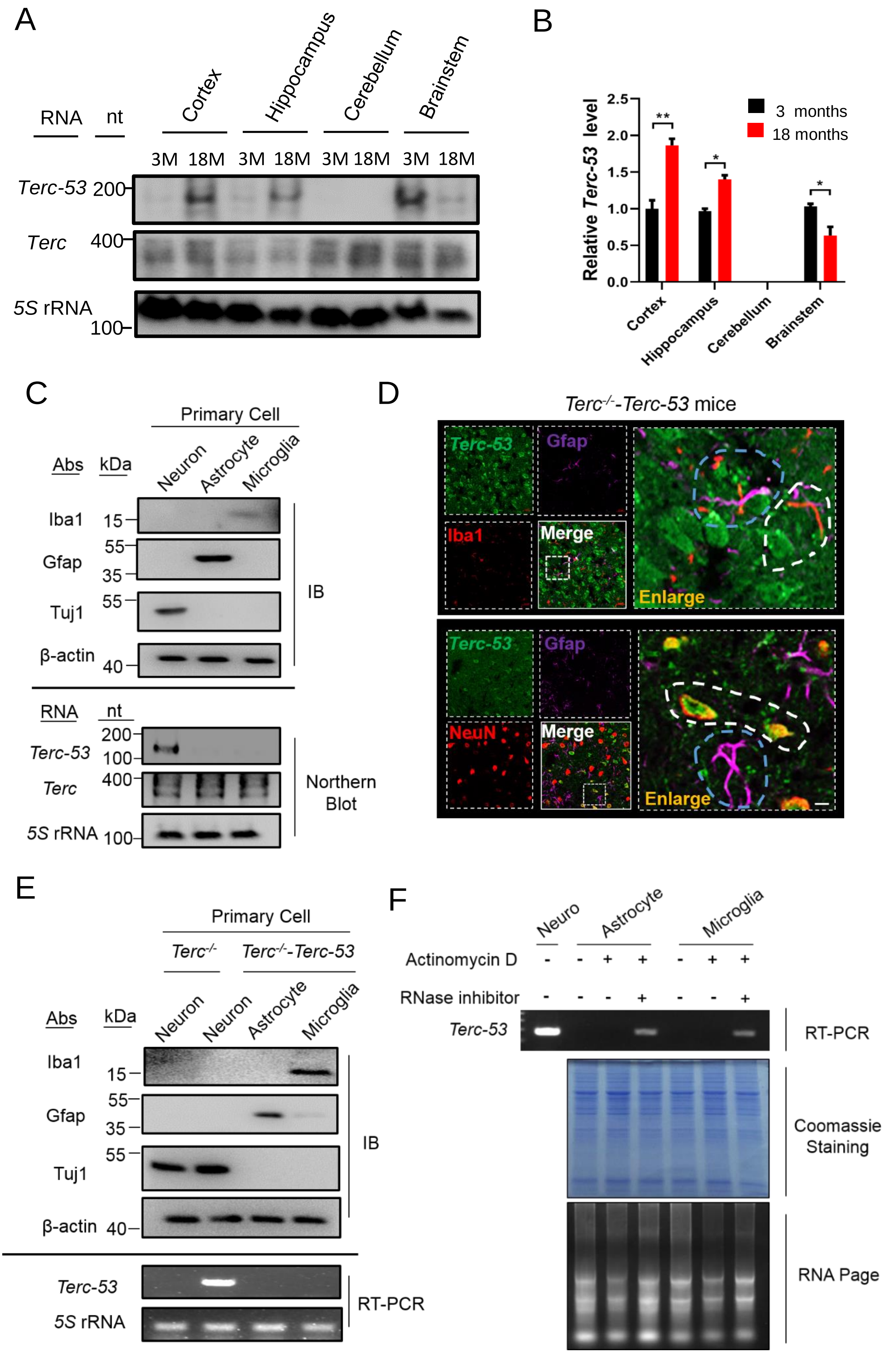
A
RNA
nt
Cortex
Hippocampus
Cerebellum
Brainstem
3M 18M 3M 18M 3M 18M 3M 18M
Terc-53 200
Terc 400
5S rRNA 100
B
Relative Terc-53 level
3 months
18 months
Cortex
Hippocampus
Cerebellum
Brainstem
C
Primary Cell
Abs kDa
Neuron
Astrocyte
Microglia
Iba1 15
Gfap 55 35
Tuj1 55
β-actin 40
IB
RNA nt
Terc-53 200 100
Terc 400
5S rRNA 100
Northern Blot
D
Terc-/--Terc-53 mice
Terc-53
Gfap
Iba1
Merge
Enlarge
NeuN
E
Primary Cell
Terc-/-
Terc-/--Terc-53
Abs kDa
Neuron
Neuron
Astrocyte
Microglia
Iba1 15
Gfap 55 35
Tuj1 55
β-actin 40
IB
Terc-53
5S rRNA
RT-PCR
F
Neuro
Astrocyte
Microglia
Actinomycin D
RNase inhibitor
Terc-53
RT-PCR
Coomassie Staining
RNA Page

Figure S6. ***Terc-53*** **is specifically expressed in neurons.**

(A) *Terc-53* and full-length *Terc* expression levels in different brain regions of 3-month-old and 18-month-old wild-type male mice were examined by Northern blotting. *5S* rRNA was used as a loading control.

(B) Quantification of the relative *Terc-53* levels in Panel A (n = 4).

(C) Top panels: isolation of primary cells from 3-month-old wild-type mice and verification of their identities using specific markers (neuron: Tuj1; astrocyte: Gfap; microglia: Iba1). β-actin was used as a loading control. Bottom panels: Northern blotting examination of *Terc-53* and full-length *Terc* levels in these primary cells. *5S* rRNA was used as a loading control.

(D) Fluorescence in situ hybridization (FISH) examination of *Terc-53* and immunofluorescence staining of Iba1, Gfap and NeuN in the hippocampus of 6-month-old *Terc*$^{-/-}$-*Terc-53* mice.

(E) Top panels: isolation of primary cells from 6-month-old *Terc*$^{-/-}$ and *Terc*$^{-/-}$-*Terc-53* mice and verification of their identities using specific markers (neuron: Tuj1; astrocyte: Gfap; microglia: Iba1). β-actin was used as a loading control. Bottom panels: RT-PCR examination of *Terc-53* levels in these primary cells. *5S* rRNA was used as a loading control.

(F) RT-PCR examination of *Terc-53* levels in *Terc*$^{-/-}$-*Terc-53* primary neurons, astrocytes and microglia after actinomycin D (an transcription inhibitor) or/and RNase inhibitor treatment. The cells were treated with actinomycin D (10 μg/ml) and RNase inhibitors (100 U/ml) for 2 h and 1 h, respectively. Bottom panels are a Coomassie staining gel as the loading control and an agarose gel of the total RNA as the inhibitor treatment control.

Data are presented as Mean ± SD. ns, no significance, *p < 0.05, **p < 0.01.

Figure S7

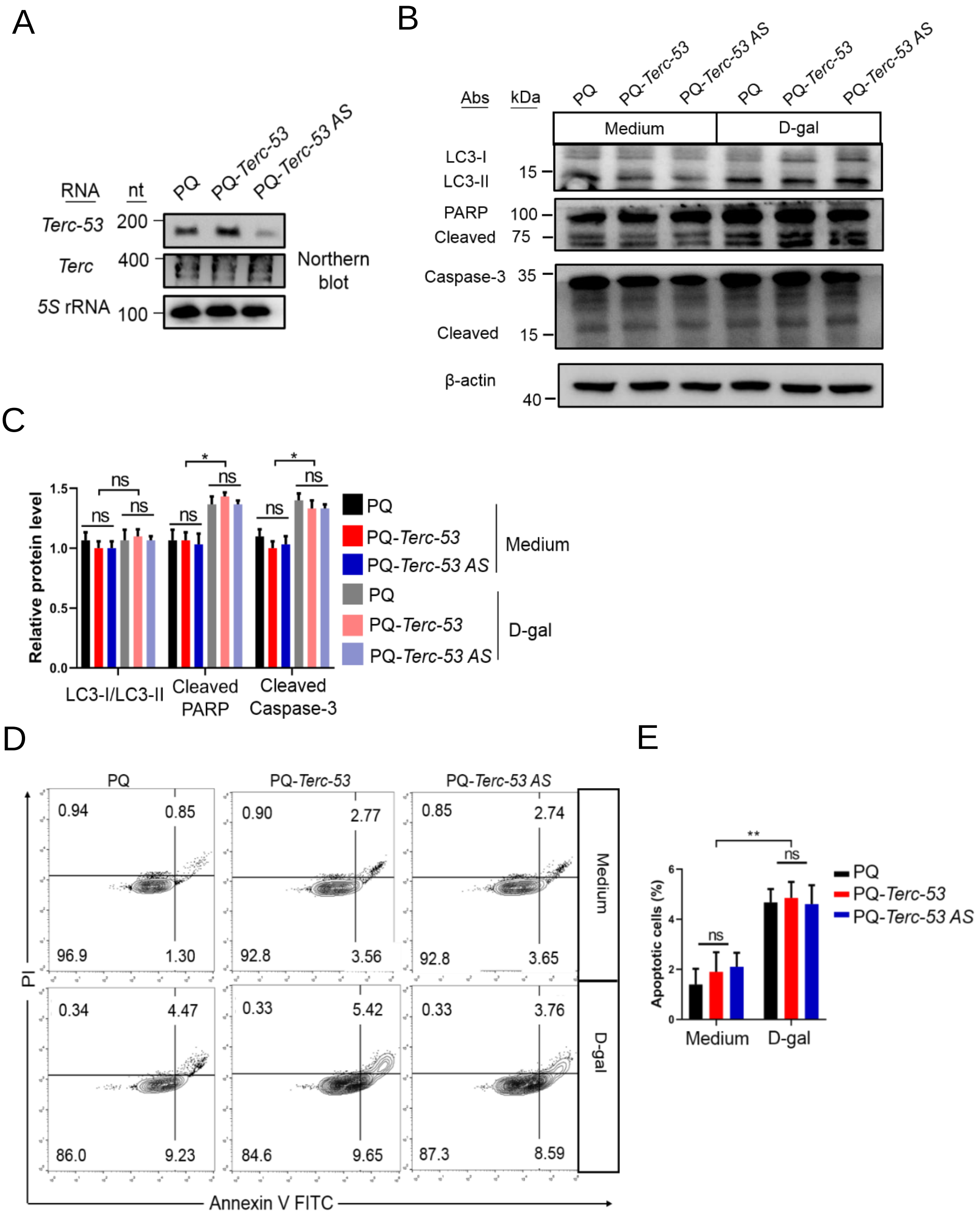

A
RNA
nt
PQ
PQ-Terc-53
PQ-Terc-53 AS
Terc-53
200
Terc
400
5S rRNA
100
Northern blot
B
Abs
kDa
PQ
PQ-Terc-53
PQ-Terc-53 AS
PQ
PQ-Terc-53
PQ-Terc-53 AS
Medium
D-gal
LC3-I
LC3-II
15
PARP
Cleaved
100
75
Caspase-3
35
Cleaved
15
β-actin
40
C
Relative protein level
1.5
1.0
0.5
0.0
ns
*
LC3-I/LC3-II
Cleaved PARP
Cleaved Caspase-3
PQ
PQ-Terc-53
PQ-Terc-53 AS
Medium
PQ
PQ-Terc-53
PQ-Terc-53 AS
D-gal
D
PQ
PQ-Terc-53
PQ-Terc-53 AS
0.94
0.85
96.9
1.30
0.90
2.77
92.8
3.56
0.85
2.74
92.8
3.65
Medium
0.34
4.47
86.0
9.23
0.33
5.42
84.6
9.65
0.33
3.76
87.3
8.59
D-gal
PI
Annexin V FITC
E
Apoptotic cells (%)
6
4
2
0
**
ns
Medium
D-gal
PQ
PQ-Terc-53
PQ-Terc-53 AS

Figure S7. *Terc-53* does not affect autophagy or apoptosis of N2a cells.

(A) Northern blotting examination of *Terc-53* and full-length *Terc* levels in *Terc-53*-overexpressing (*Terc-53*), *Terc-53* antisense-expressing (*Terc-53 AS*), or the vector control (PQ) N2a cells. *5S* rRNA was used as a loading control.

(B) Immunoblotting examination of of LC3, cleaved-PARP, Caspase-3 and β-actin in *Terc-53*-overexpressing (*Terc-53*), *Terc-53* antisense-expressing (*Terc-53 AS*), or the vector control (PQ) N2a cells, with or without D-galactose (D-gal) treatment.

(C) Quantification of the relative protein levels in Panel B (n = 3).

(D) Flow cytometry examination of apoptosis rates of *Terc-53*-overexpressing (*Terc-53*), *Terc-53* antisense-expressing (*Terc-53 AS*), or the vector control (PQ) N2a cells, with or without D-galactose (D-gal) treatment. FITC-conjugated Annexin V (AV)/PI double-staining was used to label the apoptotic cells.

(E) Quantification of apoptotic cells in Panel D (n = 3).

Data are presented as mean ± SD. ns, no significance, $*p < 0.05$, $**p < 0.01$.

Figure S8

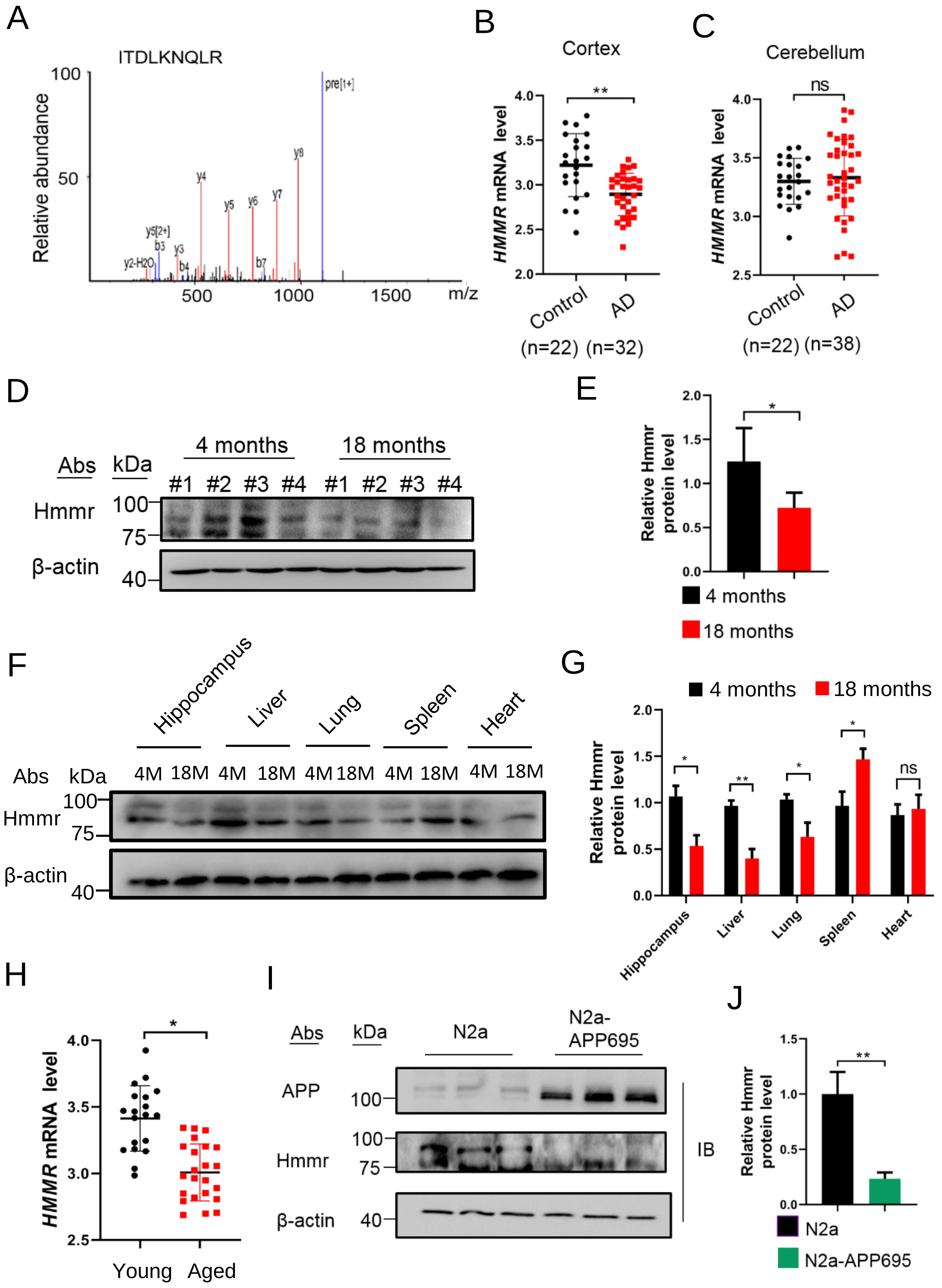
A
ITDLKNQLR
Relative abundance
100
50
pre[1+]
y8
y4
y7
y5
y6
y5[2+]
b3
y3
y2-H2O
b4
b7
500
1000
1500
m/z
B
Cortex
HMMR mRNA level
**
Control
AD
(n=22) (n=32)
C
Cerebellum
ns
Control
AD
(n=22) (n=38)
D
4 months
18 months
Abs
kDa
#1 #2 #3 #4 #1 #2 #3 #4
Hmmr
β-actin
100
75
40
E
Relative Hmmr protein level
*
4 months
18 months
F
Hippocampus
Liver
Lung
Spleen
Heart
Abs
kDa
4M 18M 4M 18M 4M 18M 4M 18M 4M 18M
Hmmr
β-actin
G
4 months
18 months
Relative Hmmr protein level
ns
Hippocampus
Liver
Lung
Spleen
Heart
H
HMMR mRNA level
*
Young
Aged
I
Abs
kDa
N2a
N2a-APP695
APP
Hmmr
β-actin
IB
J
Relative Hmmr protein level
**
N2a
N2a-APP695

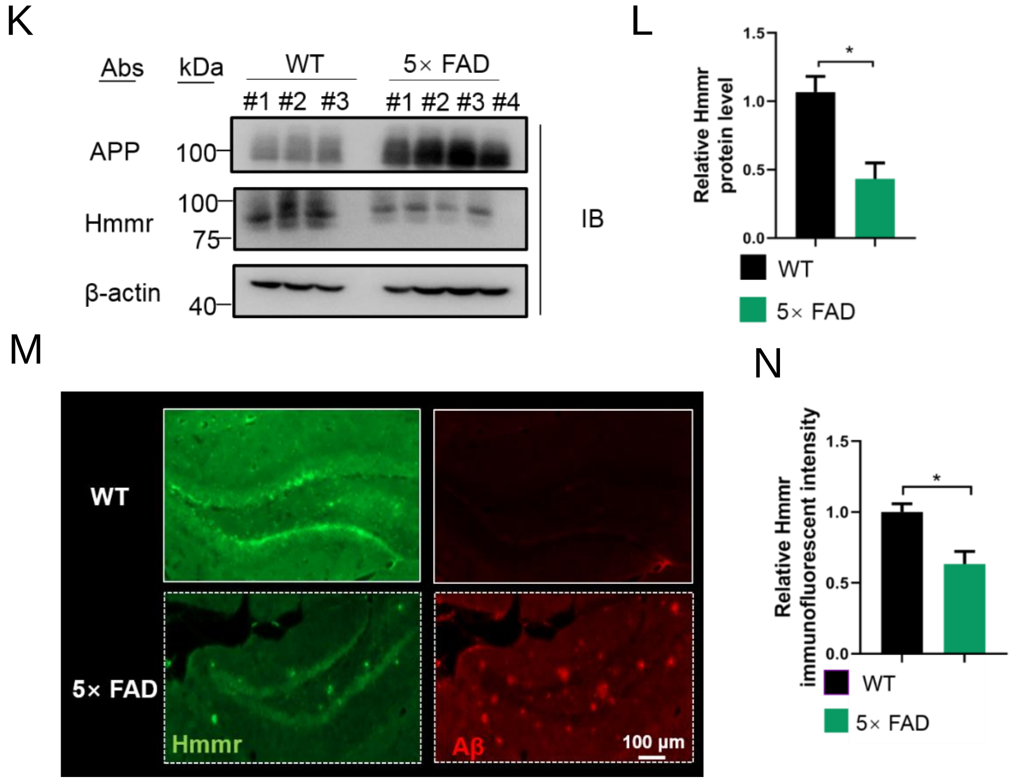
K
Abs
kDa
WT
5× FAD
#1 #2 #3
#1 #2 #3 #4
APP
100
Hmmr
100
75
β-actin
40
IB
L
1.5
1.0
0.5
0.0
*
Relative Hmmr protein level
WT
5× FAD
M
WT
5× FAD
Hmmr
Aβ
100 μm
N
1.5
1.0
0.5
0.0
*
Relative Hmmr immunofluorescent intensity
WT
5× FAD

Figure S8. Identification of Hmmr as a potential *Terc-53* effector.

(A) Mass spectrometry identification of Hmmr purified with the procedure shown in Fig. 2b.

(B-C) *HMMR* expression in patients with Alzheimer's disease (AD) (n = 32) and unaffected controls (n = 22). Data from a tissue with AD neuropathology (cortex) and one partially spared by the disease (cerebellum) were analyzed. The sets of data were extracted from an existing database (Gene Expression Omnibus (GEO) accession number GSE118553).

(D) Immunoblotting examination of Hmmr protein levels in the hippocampus of wild-type mice at 4 months and 18 months of age.

(E) Quantification of the relative Hmmr protein levels in Panel D.

(F) Immunoblotting examination of Hmmr protein levels in different tissues of of wild-type male mice at 4 months and 18 months of age. β-actin were used as a loading control.

(G) Quantification of the relative Hmmr protein levels in Panel F (n = 4).

(H) *HMMR* expression levels in young and aged human brains (young < 60 years of age, n = 19; old > 60 years of age, n = 23), revealed by analyzing an existing RNAseq database (Gene Expression Omnibus (GEO) accession number GSE118553).

(I) Immunoblotting examination of APP and Hmmr protein levels in N2a and N2a-APP695 cells. β-actin were used as a loading control.

(J) Quantification of the relative Hmmr protein levels in Panel I (n = 3).

(K) Immunoblotting examination of Hmmr and APP protein levels in the hippocampus of 5× FAD male mice and wild-type (WT) male mice at 6 months of age. β-actin were used as a loading control.

(L) Quantification of the relative Hmmr protein levels in Panel K (n = 3).

(M) Immunofluorescent staining of Hmmr and Aβ in the hippocampus of 6-month-old WT and 5× FAD male mice.

(N) Quantification of Hmmr protein levels in Panel M (n = 3).

Data are presented as Mean ± SD. ns, no significance, *$p < 0.05$, **$p < 0.01$

Figure S9

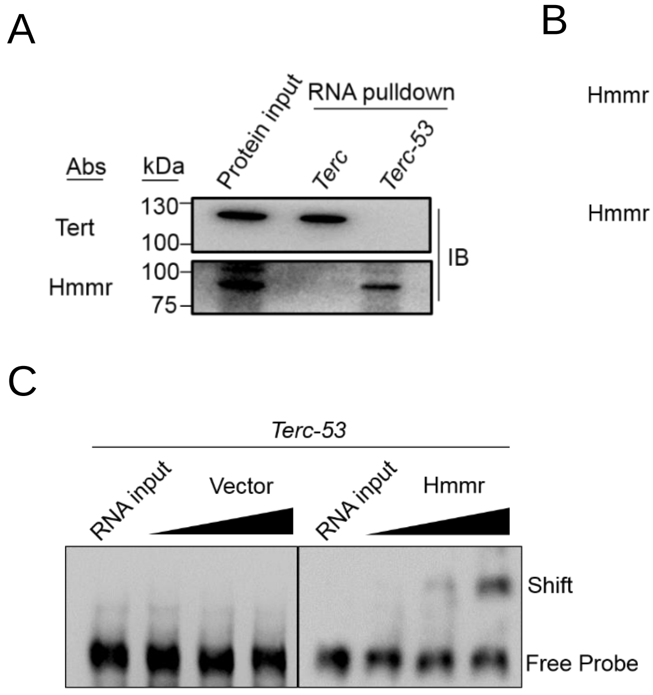

Figure S9. Hmmr physically interacts with *Terc-53*.

(A) Biotinylated *Terc-53* or full-length *Terc* was incubated with N2a cytosolic lysates for the RNA pulldown assay, followed by immunoblotting of the indicated proteins.

(B) Coomassie staining of purified Hmmr (indicated by the red star); the concentration was determined using the BSA standards on the right. The purified protein was also verified by immunoblotting (Top left).

(C) Gel shift assay of biotin-labeled *Terc-53* with in vitro purified Hmmr and the vector-control eluate.

Figure S10

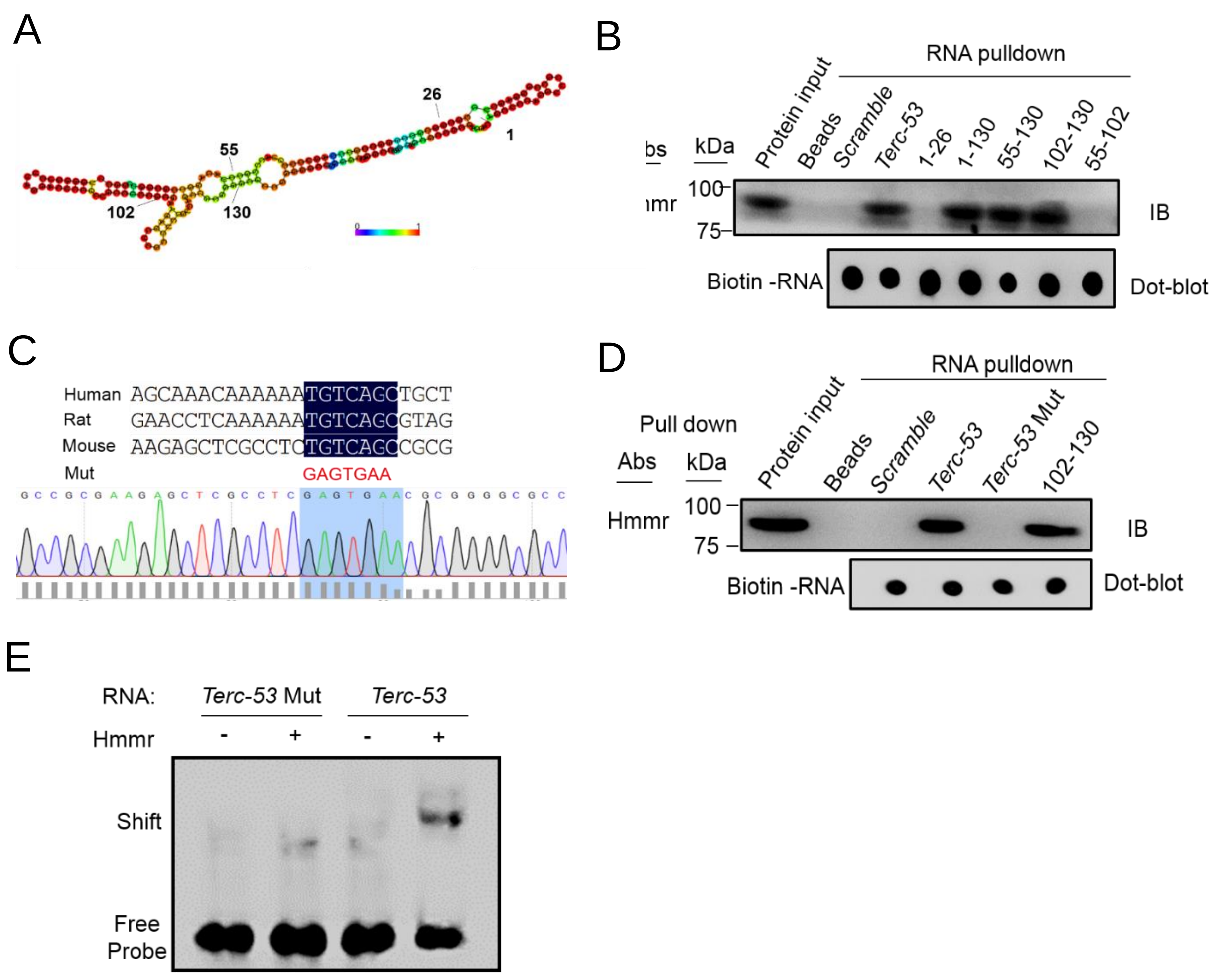

Figure S10. Nucleotides 102-130 of *Terc-53* are involved in interaction with Hmmr.

(A) The predicted secondary structure of *Terc-53* by the Vienna RNA Package. The prediction was based on minimum free energy (MFE). Color scale indicates confidence of prediction for each base. Red shades indicate the predictions with strong confidence.

(B) In vitro RNA/protein interaction assay between biotin-labeled *Terc-53* of different lengths and purified His-Hmmr protein.

(C) The sequences of human, rat and mouse *Terc-53* (nt 102-130) with the most conserved region highlighted in black, and the changed nucleotides in mouse *Terc-53* mutant in red. The bottom panel shows the Sanger sequencing result, verifying the construction of the mutant.

(D) In vitro RNA/protein interaction assay between purified His-Hmmr protein, and the biotin-labeled RNAs as indicated. *Terc-53* and *Terc-53* 102-130 were used as positive controls.

(E) Gel shift assay of biotin-labeled *Terc-53* or *Terc-53* Mutant with purified His-Hmmr protein.

Figure S11

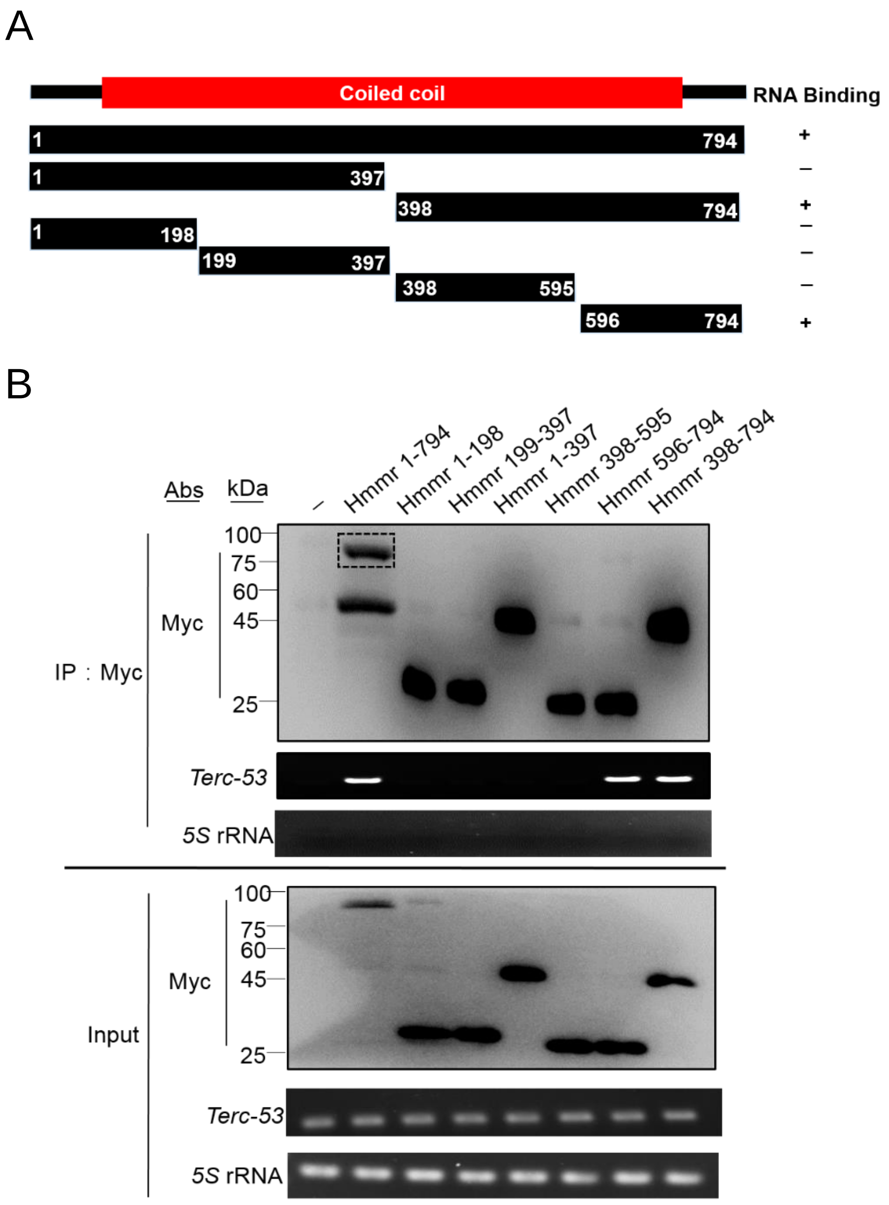

Figure S11. The C-terminus (amino acids 596-794) of Hmmr is involved in interaction with *Terc-53*.

(A) Schematic of Hmmr and different truncations.

(B) N2a cells were transfected with full-length or truncation constructs of Myc-Hmmr for 2 days. Hmmr complexes were immuno-precipitated using Myc magnetic beads, and examined by immunoblotting using anti-Myc antibody. The box of dashed line indicates the band of the correct size of Hmmr 1-794. The levels of co-purified *Terc-53* were determined by RT-PCR.

Figure S12

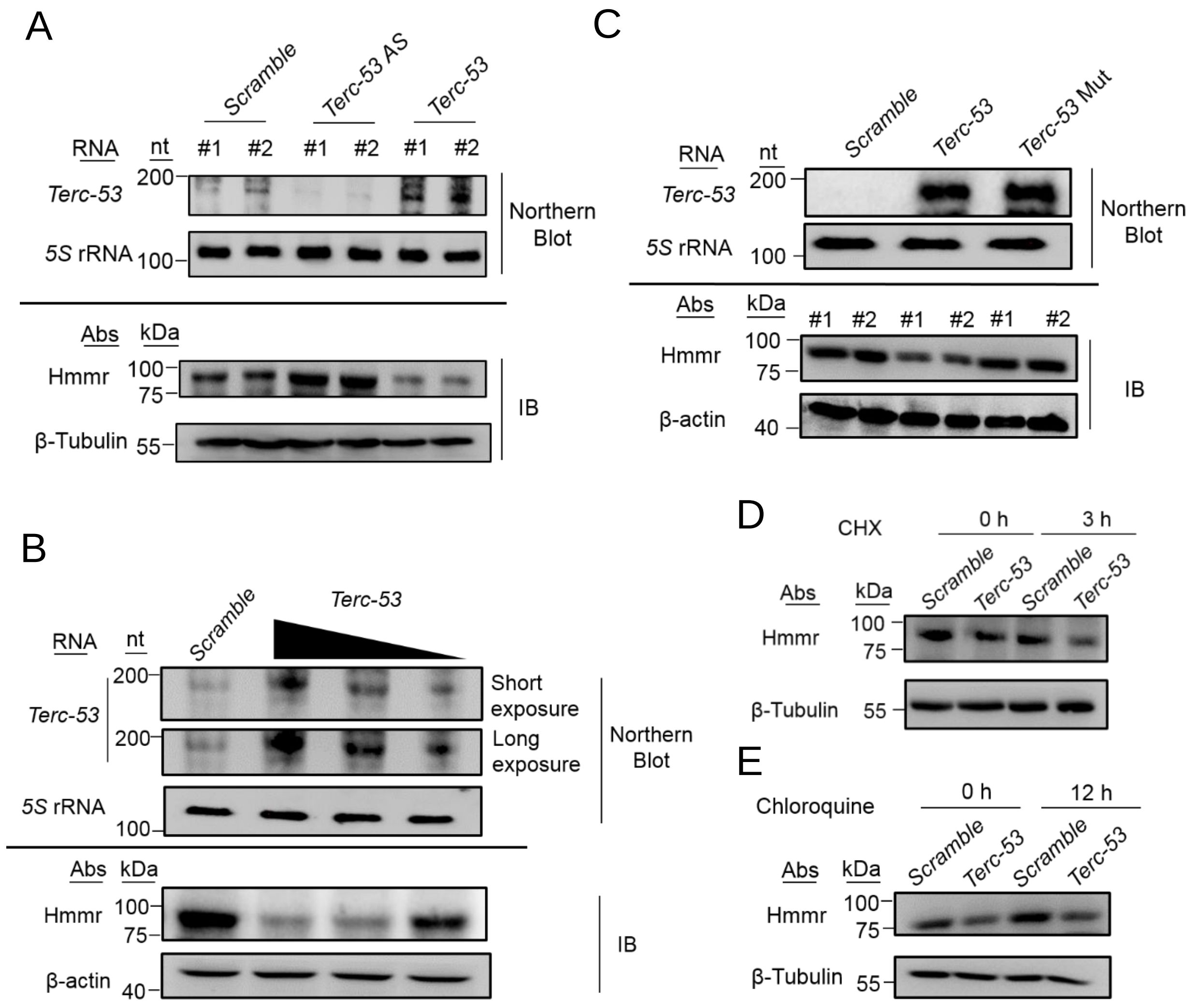
A
Scramble
Terc-53 AS
Terc-53
RNA
nt
#1
#2
Terc-53
200
5S rRNA
100
Northern Blot
Abs
kDa
Hmmr
100
75
β-Tubulin
55
IB
B
Scramble
Terc-53
Short exposure
Long exposure
Northern Blot
β-actin
40
C
Terc-53 Mut
D
CHX
0 h
3 h
E
Chloroquine
12 h

Figure S12. ***Terc-53*** **regulates Hmmr protein stability.**

(A) Northern blotting examination of *Terc-53* levels (Top) and immunoblotting examination of Hmmr protein levels (Bottom) in N2a cells stably expressing *Terc-53*, *Terc-53 AS* or the *Scramble* RNA. *5S* rRNA and β-tubulin were used as loading controls.

(B) Immunoblotting examination of Hmmr protein levels (bottom panels) in N2a cells expressing different levels of *Terc-53* or the *Scramble* RNA. *Terc-53* levels were monitored by Northern blotting (top panels). *5S* rRNA and β-actin were used as loading controls.

(C) Immunoblotting examination of Hmmr protein levels (bottom panels) in N2a cells expressing *Terc-53*, *Terc-53* Mut that does not bind Hmmr, or the *Scramble* RNA. *Terc-53* and *Terc-53* Mut levels were monitored by Northern blotting (top panels). *5S* rRNA and β-actin were used as loading controls.

(D) Immunoblotting examination of Hmmr proteins in N2a cells expressing *Terc-53*, or the *Scramble* RNA, before and after Cycloheximide (a protein synthesis inhibitor) treatment. β-tubulin was used as a loading control.

(E) Immunoblotting examination of Hmmr proteins in N2a cells expressing *Terc-53*, or the *Scramble* RNA, before and after Chloroquine (a autophagy inhibitor) treatment. β-tubulin was used as a loading control.

Figure S13

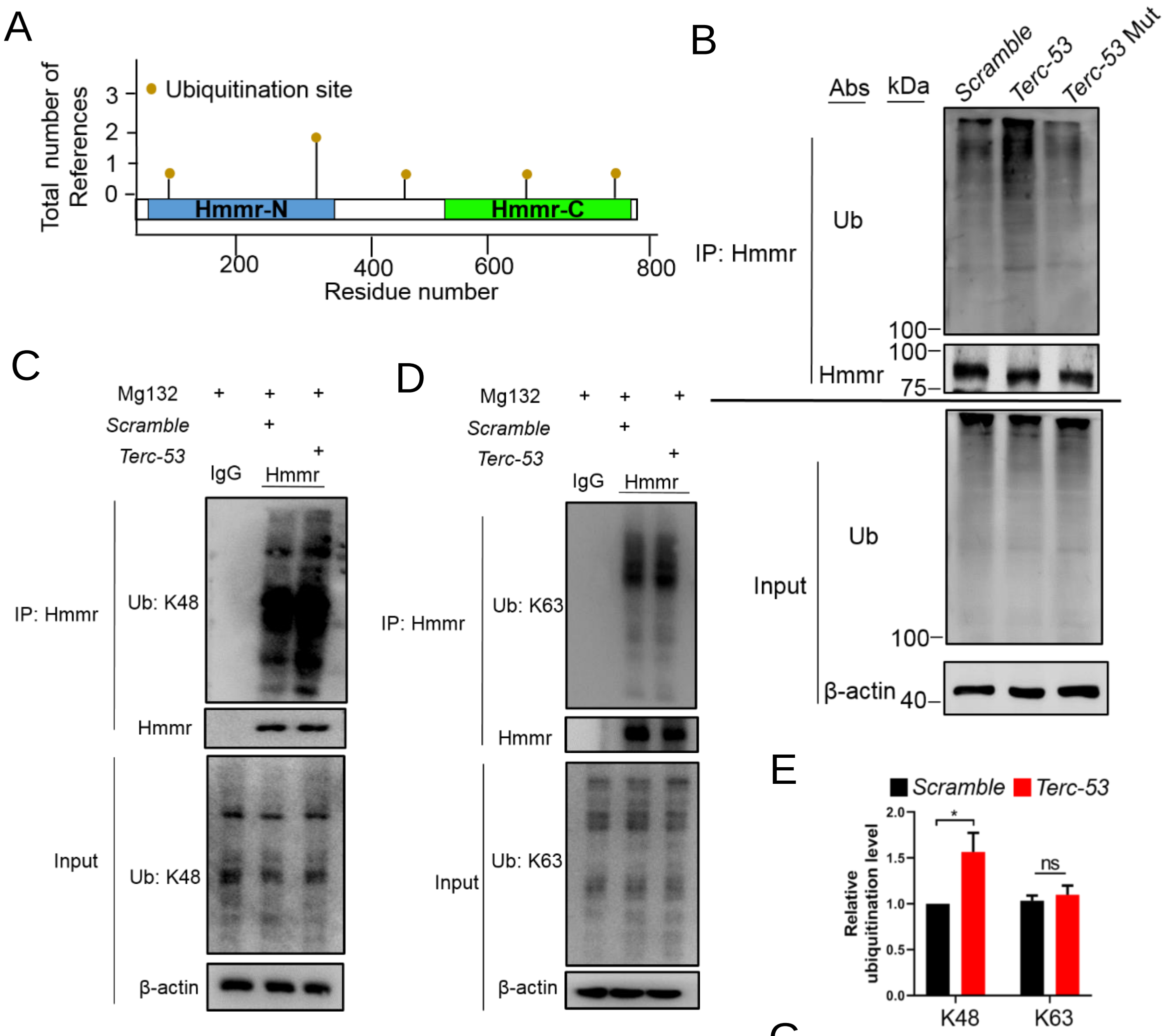

	T(U)	C	A	G	Total
Terc-53 (171 nt)	10.5%	39.2%	9.9%	40.4%	100%

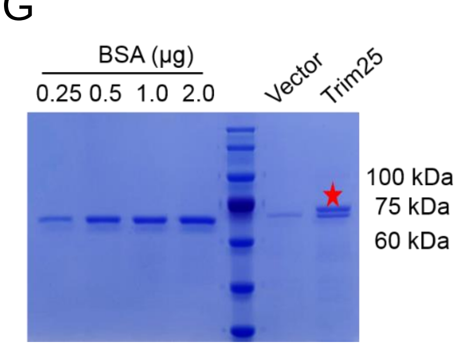

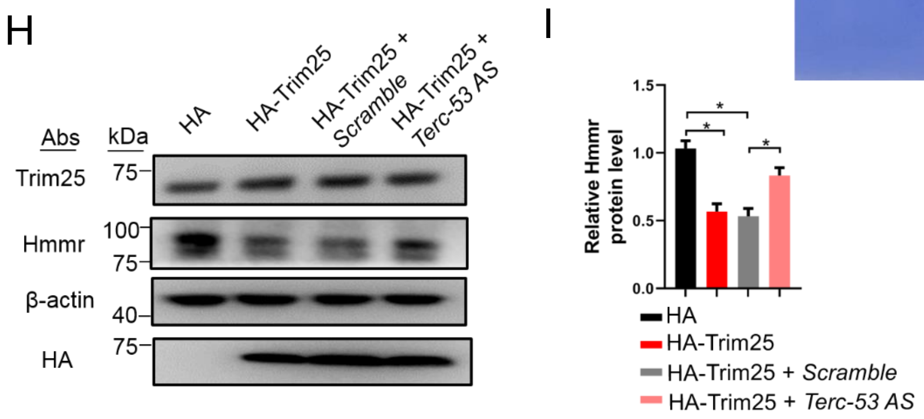

Figure S13. Trim25 is the ubiquitin ligase for *Terc-53*-mediated Hmmr degradation.

(A) The ubiquitination sites in Hmmr protein, predicted by PhosphoSitePlus.

(B) Endogenous Hmmr was immunoprecipitated from N2a cells stably expressing *Terc-53*, *Terc-53* Mut that does not bind Hmmr, or the *Scramble* RNA. The ubiquitination levels of the cell lysates (bottom) and the purified Hmmr (top) were examined by immunoblotting using a ubiquitin antibody. β-actin was used as a loading control for the cell lysate samples. The levels of purified Hmmr were monitored using a Hmmr antibody.

(C-D) Hmmr was immunoprecipitated from *Terc-53*-overexpressing (*Terc-53*) or the *Scramble* RNA-expressing (*Scramble*) N2a cells. The purified proteins were examined with an antibody specific for K48-linked (Ub: K48) (Panel G) or K63-linked (Ub: K63) (Panel H) polyubiquitin chains.

(E) Quantification of the relative ubiquitination levels in Panel C and D.

(F) GC content enrichment analysis of *Terc-53*.

(G) Coomassie staining of purified HA-Trim25 (indicated by the red star). The concentration of the purified protein was determined using BSA standards.

(H) Immunoblotting examination of Hmmr protein levels in N2a cells expressing *Terc-53* antisense RNA (*Terc-53 AS*) or the *Scramble* RNA in addition to overexpressing Trim25.

(I) Quantification of the relative Hmmr protein levels in Panel H ($n = 3$).

Data are presented as mean ± SD. ns, no significance, *$p < 0.05$, **$p < 0.01$.

Figure S14

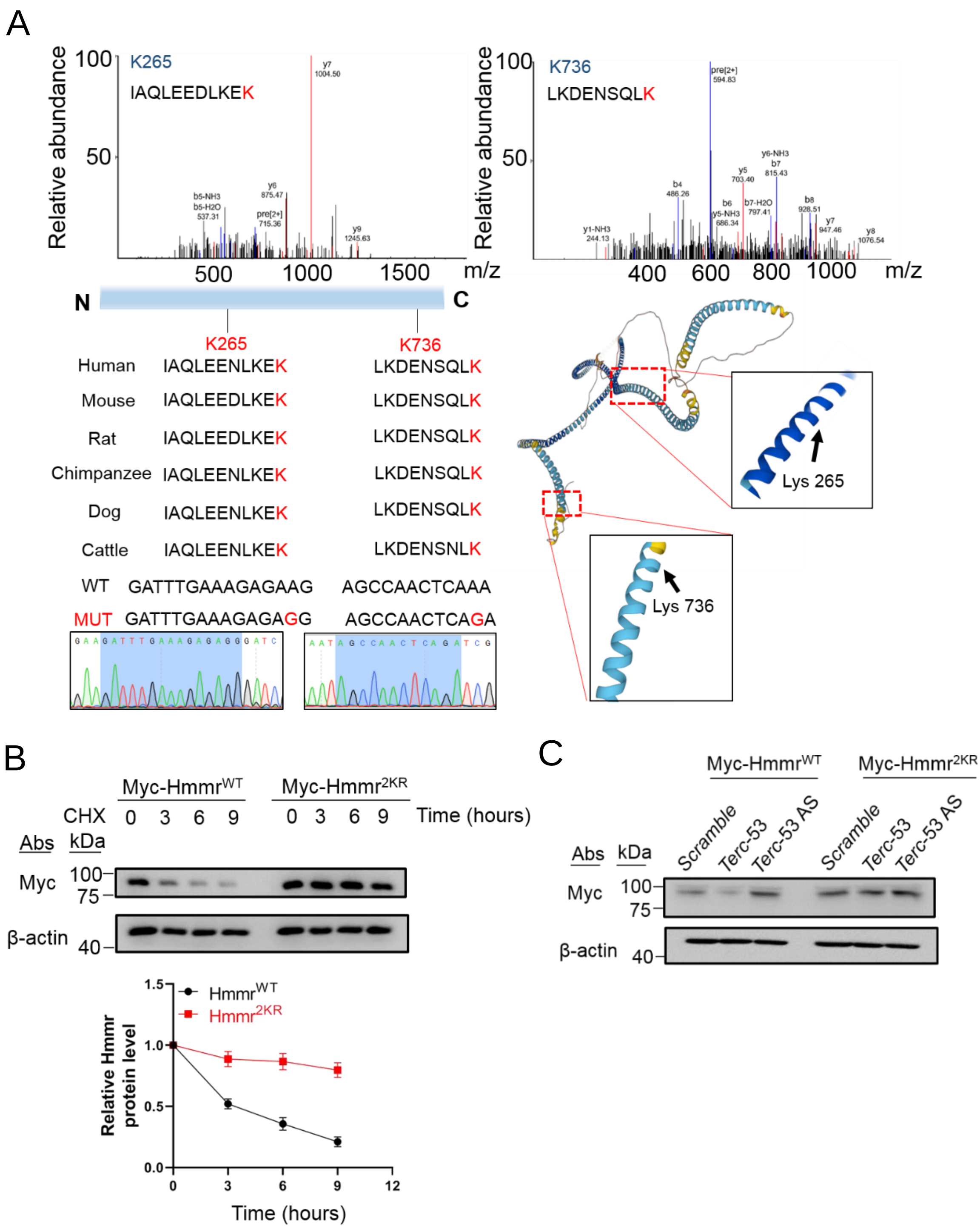

A
Relative abundance
K265
IAQLEEDLKEK
m/z
K736
LKDENSQLK
N
C
K265
K736
Human IAQLEENLKEK LKDENSQLK
Mouse IAQLEEDLKEK LKDENSQLK
Rat IAQLEEDLKEK LKDENSQLK
Chimpanzee IAQLEENLKEK LKDENSQLK
Dog IAQLEENLKEK LKDENSQLK
Cattle IAQLEENLKEK LKDENSNLK
WT GATTTGAAAGAGAAG AGCCAACTCAAA
MUT GATTTGAAAGAGAGG AGCCAACTCAGA
Lys 265
Lys 736
B
Myc-Hmmr^WT
Myc-Hmmr^2KR
CHX 0 3 6 9 0 3 6 9 Time (hours)
Abs kDa
Myc
β-actin
HmmrWT
Hmmr2KR
Relative Hmmr protein level
Time (hours)
C
Myc-Hmmr^WT
Myc-Hmmr^2KR
Scramble
Terc-53
Terc-53 AS
Abs kDa
Myc
β-actin

Figure S14. Mutation of the ubiquitination sites on Hmmr stabilizes the protein.

(A) HEK293T cells were co-transfected with HA-tagged Trim25, Flag-tagged ubiquitin, and Myc-tagged Hmmr. Cell lysates were subjected to immunoprecipitation and Mass spectrometry analysis to identify ubiquitination sites. The Hmmr peptides with a ubiquitination site were highlighted in red (upper panels). Lower panels: A schematic diagram showing the two evolutionarily conserved lysine residues (K265 and K736) for Hmmr ubiquitination, and the protein structure predict by Alphafold2.

(B) N2a cells expressing Myc-tagged wild-type Hmmr (Myc-Hmmr^{WT}) or the K265R+K736R mutant (Myc-Hmmr^{2KR}) were treated with cycloheximide (CHX) (a translation inhibitor) for the indicated periods of time. Lysates were subjected to immunoblotting analysis with Myc and β-actin antibodies (top panels). The bottom panel: quantification of the relative levels of Myc-Hmmr^{WT} and Myc-Hmmr^{2KR} with time (n = 3).

(C) N2a cells stably expressing *Terc-53* and *Terc-53 AS* or the *Scramble* RNA were transfected with Myc-Hmmr^{WT} or Myc-Hmmr^{2KR}. The expression levels of Myc-Hmmr^{WT} or Myc-Hmmr^{2KR} were examining by immunoblotting.

Data are presented as mean ± SD. ns, no significance, *$p < 0.05$, **$p < 0.01$.

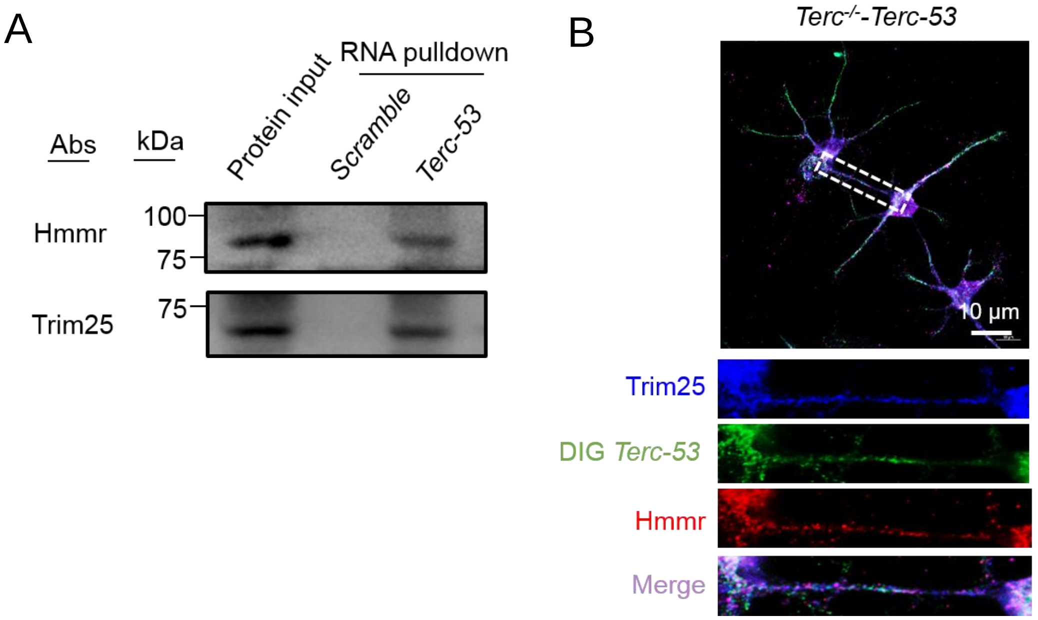

Figure S15. Interaction of *Terc-53*, Hmmr and Trim25 in primary neurons.

(A) Biotinylated *Terc-53* or the *Scramble* RNA were incubated with the lysate of 16-day DIV (days in vitro) *Terc*$^{-/-}$ primary neurons for the RNA pulldown assay, followed by immunoblotting of the indicated proteins.

(B) Representative images of *Terc-53* FISH, and Hmmr and Trim25 immunostaining in *Terc*$^{-/-}$-*Terc-53* primary neurons.

Figure S16

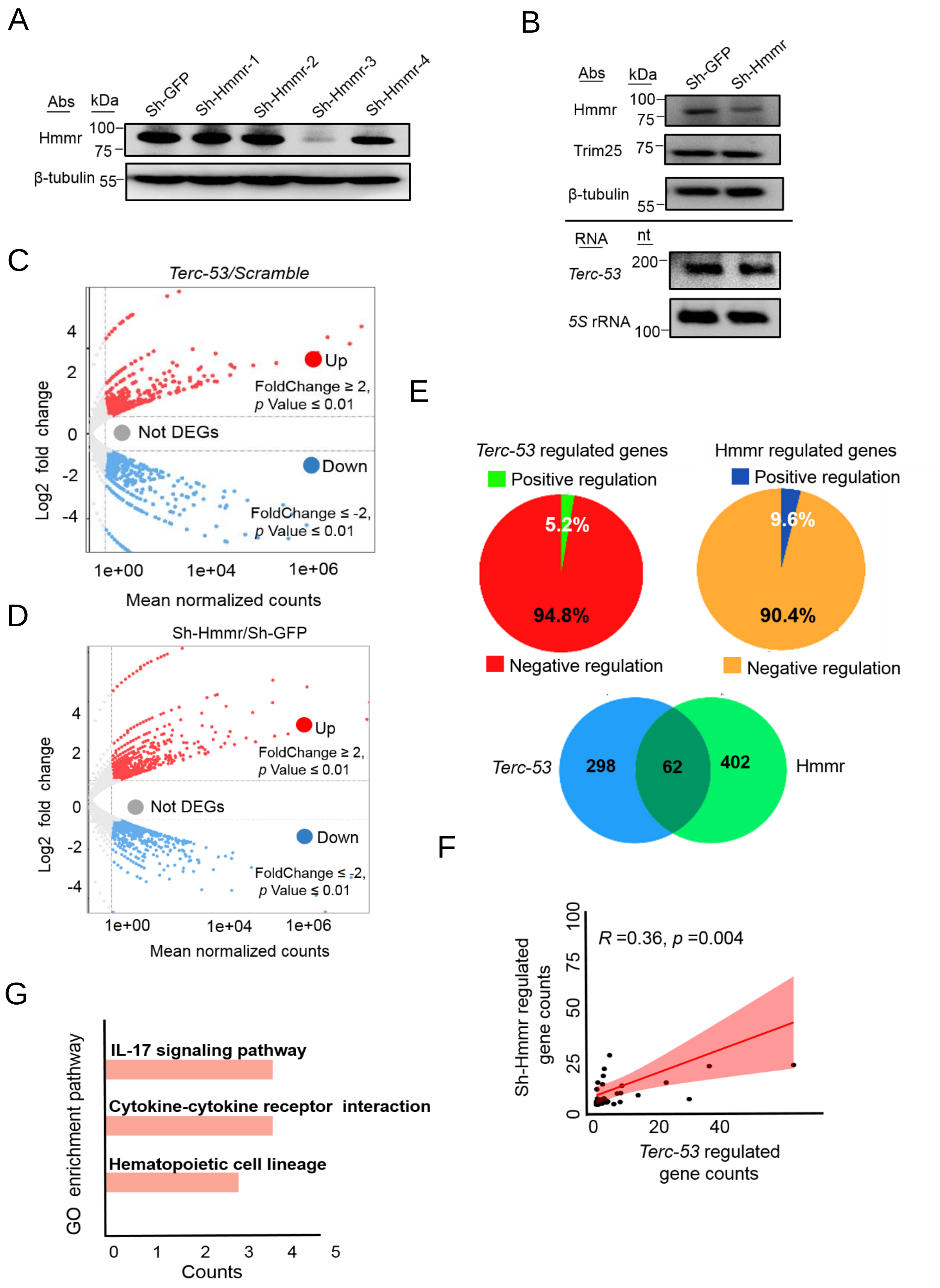

A
Abs
kDa
Sh-GFP
Sh-Hmmr-1
Sh-Hmmr-2
Sh-Hmmr-3
Sh-Hmmr-4
Hmmr
100
75
β-tubulin
55
B
Abs
kDa
Sh-GFP
Sh-Hmmr
Hmmr
100
75
Trim25
75
β-tubulin
55
RNA
nt
Terc-53
200
5S rRNA
100
C
Terc-53/Scramble
Up
FoldChange ≥ 2,
p Value ≤ 0.01
Not DEGs
Down
FoldChange ≤ -2,
p Value ≤ 0.01
Log2 fold change
Mean normalized counts
1e+00
1e+04
1e+06
D
Sh-Hmmr/Sh-GFP
Up
FoldChange ≥ 2,
p Value ≤ 0.01
Not DEGs
Down
FoldChange ≤ -2,
p Value ≤ 0.01
Log2 fold change
Mean normalized counts
E
Terc-53 regulated genes
Positive regulation
5.2%
94.8%
Negative regulation
Hmmr regulated genes
Positive regulation
9.6%
90.4%
Negative regulation
Terc-53
298
62
402
Hmmr
F
R =0.36, p =0.004
Sh-Hmmr regulated gene counts
Terc-53 regulated gene counts
G
IL-17 signaling pathway
Cytokine-cytokine receptor interaction
Hematopoietic cell lineage
GO enrichment pathway
Counts

Figure S16. RNA-seq analysis of *Terc-53*-overexpressing or Hmmr-knockdown N2a cells.

(A) Immunoblotting examination of Hmmr protein levels in N2a cells with different shRNA constructs for Hmmr knockdown. A shRNA construct for GFP (sh-GFP) was used as a negative control. β-tubulin was used as a loading control.

(B) The levels of Trim25 and *Terc-53* in N2a cells with (sh-Hmmr) or without (sh-GFP) Hmmr knockdown.

(C) Differentially expressed genes (DEGs) identified from comparison of the expression profile in *Terc-53*-overexpressing N2a cells and that in the *Scramble* RNA-expressing cells. Red dots represent upregulated DEGs. Blue dots represent downregulated DEGs. Gray dots represent non-DEGs.

(D) Differentially expressed genes (DEGs) identified from comparison of the expression profile of Hmmr-knockdown (sh-Hmmr) N2a cells and that of the control (sh-GFP) cells. Red dots represent upregulated DEGs. Blue dots represent downregulated DEGs. Gray dots represent non-DEGs.

(E) Venn diagram showing the overlap of transcripts from positively-regulated and negatively-regulated gene sets.

(F) Correlation analysis of differentially expressed genes in *Terc-53*-overexpressing or Hmmr-knockdown cells normalized to control. P values were calculated by Fisher's exact test.

(G) Gene ontology (GO) pathway analysis of genes co-regulated by *Terc-53* overexpression and Hmmr knockdown.

Figure S17

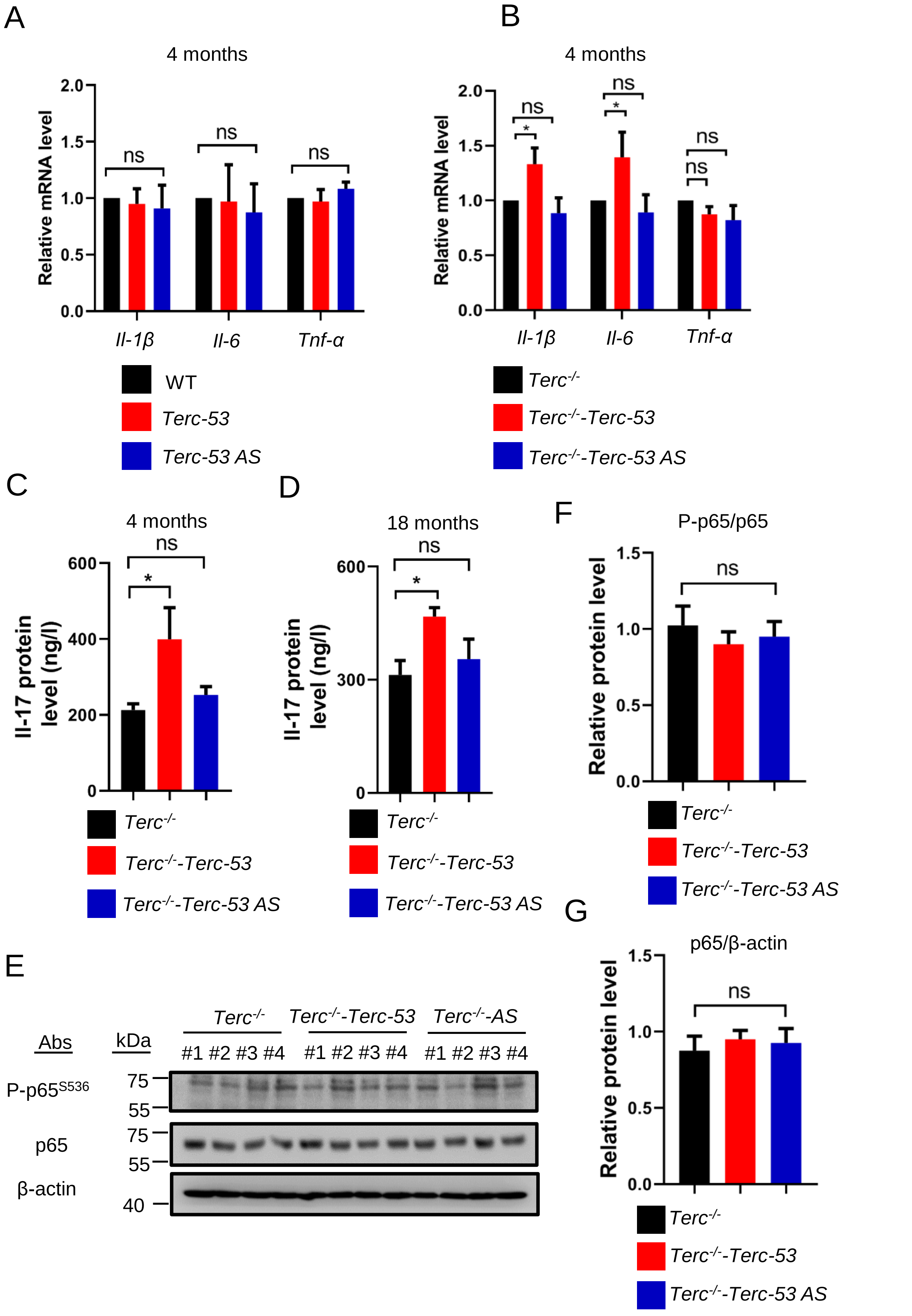
A
4 months
Relative mRNA level
2.0
1.5
1.0
0.5
0.0
ns
ns
ns
Il-1β
Il-6
Tnf-α
WT
Terc-53
Terc-53 AS
B
4 months
Relative mRNA level
2.0
1.5
1.0
0.5
0.0
ns
*
ns
*
ns
ns
Il-1β
Il-6
Tnf-α
Terc-/-
Terc-/--Terc-53
Terc-/--Terc-53 AS
C
4 months
ns
*
Il-17 protein level (ng/l)
600
400
200
0
Terc-/-
Terc-/--Terc-53
Terc-/--Terc-53 AS
D
18 months
ns
*
Il-17 protein level (ng/l)
600
300
0
Terc-/-
Terc-/--Terc-53
Terc-/--Terc-53 AS
F
P-p65/p65
Relative protein level
1.5
1.0
0.5
0.0
ns
Terc-/-
Terc-/--Terc-53
Terc-/--Terc-53 AS
E
Abs
kDa
Terc-/-
Terc-/--Terc-53
Terc-/--AS
#1 #2 #3 #4
#1 #2 #3 #4
#1 #2 #3 #4
P-p65S536
75
55
p65
75
55
β-actin
40
G
p65/β-actin
Relative protein level
1.5
1.0
0.5
0.0
ns
Terc-/-
Terc-/--Terc-53
Terc-/--Terc-53 AS

Figure S17. The effects of *Terc-53* overexpression on pro-inflammatory factors.

(A) RT-qPCR analysis of the levels of the mRNAs as indicated in the hippocampus of WT, *Terc-53* and *Terc-53 AS* male mice at 4 months of age (n = 4).

(B) RT-qPCR analysis of the levels of the mRNAs as indicated in the hippocampus of *Terc*$^{-/-}$, *Terc*$^{-/-}$*-Terc-53* and *Terc*$^{-/-}$*-Terc-53 AS* male mice at 4 months of age (n = 4).

(C-D) Il-17 protein levels in the hippocampus of 4 months-old (Panel C) and 18-month-old (Panel D) *Terc*$^{-/-}$ and *Terc*$^{-/-}$*-Terc-53 and Terc*$^{-/-}$*-Terc-53 AS* male mice as determined by ELISA (n = 3).

(E) Immunoblots of phospho-NF-κB p65 (Ser536) (P-p65^{S536}) and p65 proteins in the hippocampus of *Terc*$^{-/-}$ and *Terc*$^{-/-}$*-Terc-53 and Terc*$^{-/-}$*-Terc-53 AS* (*Terc*$^{-/-}$*-AS*) male mice at 18 months of age. β-actin was used as a loading control.

(F-G) Quantification of the relative protein levels in Panel E (n = 4).

Data are presented as Mean ± SD. ns, no significance, *p < 0.05, **p < 0.01.
